# Overlapping SigH and SigE sigma factor regulons in *Corynebacterium glutamicum*

**DOI:** 10.3389/fmicb.2022.1059649

**Published:** 2023-02-28

**Authors:** Tobias Busche, Hana Dostálová, Lenka Rucká, Jiří Holátko, Ivan Barvík, Václav Štěpánek, Miroslav Pátek, Jörn Kalinowski

**Affiliations:** ^1^Microbial Genomics and Biotechnology, Center for Biotechnology, Bielefeld University, Bielefeld, Germany; ^2^Medical School East Westphalia-Lippe, Bielefeld University, Bielefeld, Germany; ^3^Institute of Microbiology, Academy of Sciences of the Czech Republic (ASCR), Prague, Czechia; ^4^Faculty of Mathematics and Physics, Institute of Physics, Charles University, Prague, Czechia

**Keywords:** *Corynebacterium*, sigma factor, regulon, promoter, stress, consensus sequence, transcriptional start site, RNA-seq

## Abstract

The sigma H (σ^Η^) and sigma E (σ^E^) subunits of *Corynebacterium glutamicum* RNA polymerase belong to Group 4 of sigma factors, also called extracytoplasmic function (ECF) sigma factors. Genes of the *C. glutamicum* σ^Η^ regulon that are involved in heat and oxidative stress response have already been defined, whereas the genes of the σ^E^ regulon, which is involved in cell surface stress response, have not been explored until now. Using the *C. glutamicum* RES167 strain and its derivative *C. glutamicum* Δ*cseE* with a deletion in the anti-σ^Ε^ gene, differential gene expression was analyzed by RNA sequencing. We found 296 upregulated and 398 downregulated genes in *C. glutamicum* Δ*cseE* compared to *C. glutamicum* RES167. To confirm the functional link between σ^Ε^ and the corresponding promoters, we tested selected promoters using the *in vivo* two-plasmid system with *gfp*uv as a reporter gene and by *in vitro* transcription. Analyses with RNAP+σ^Η^ and RNAP+σ^Ε^, which were previously shown to recognize similar promoters, proved that the σ^Η^ and σ^E^ regulons significantly overlap. The σ^E^-controlled genes were found to be involved for example in protein quality control (*dnaK*, *dnaJ2*, *clpB*, and *clpC*), the regulation of Clp proteases (*clgR*), and membrane integrity maintenance. The single-promoter analyses with σ^Η^ and σ^Ε^ revealed that there are two groups of promoters: those which are exclusively σ^Η^-specific, and the other group of promoters, which are σ^Η^/σ^E^-dependent. No exclusively σ^E^-dependent promoter was detected. We defined the consensus sequences of exclusively σ^Η^-regulated promotors to be −35 GGAAt and − 10 GTT and σ^Η^/σ^E^-regulated promoters to be −35 GGAAC and − 10 cGTT. Fifteen genes were found to belong to the σ^Η^/σ^Ε^ regulon. Homology modeling showed that there is a specific interaction between Met170 in σ^Η^ and the nucleotides −31 and − 30 within the non-coding strand (AT or CT) of the σ^Η^-dependent promoters. In σ^E^, Arg185 was found to interact with the nucleotides GA at the same positions in the σ^E^-dependent promoters.

## Introduction

Most bacterial genes are transcribed by the RNA polymerase (RNAP) holoenzyme that includes a primary sigma subunit (σ factor referred to as RpoD/σ^70^ in *Escherichia coli* and SigA/σ^A^ in many other bacteria) that is responsible for the transcription of housekeeping genes. Transcription of the genes involved in starvation, stationary phase, and general stress response largely depends on the presence of the primary-like σ factor usually named σ^Β^ in Gram-positive bacteria. The stress-responsive genes, which are active during various adverse environmental conditions, are in many cases transcribed by RNAP with an ECF (extracytoplasmic function) sigma factor. This group of σ factors is the most numerous and variable. The ECF sigma factors σ^H^ and σ^E^ are responsible for the transcription of large gene groups in Actinobacteria. These σ factors share some significant features in streptomycetes, mycobacteria, and corynebacteria, and probably also in rhodococci (Blumenstein et al., 2022; Štěpánek et al., 2022).

σ^Η^ controls a wide range of stress response genes in *Mycobacterium tuberculosis*, which has a portfolio of 10 ECF sigma factors. These σ^Η^-dependent genes are involved in oxidative and heat stress response, the repair of DNA damage, recovery of ribosome function, sulfur transport, and synthesis of sulfur-containing amino acids (Sharp et al., 2016). Moreover, it is required for full virulence in mice and primates (Mehra et al., 2012). In *Streptomyces coelicolor*, SigR (named R according to the redox stress), encoded by an ortholog of *M. tuberculosis* SigH, is responsible for many stress response functions, e.g., thiol homeostasis (redox control), antibiotic stress response, sulfur metabolism, ribosome modulation, energy metabolism, DNA repair, and protein turnover (Park et al., 2019). σ^Η^ in *C. glutamicum*, another member of the Actinobacteria phylum, controls similar functions: heat and oxidative stress response and DNA repair (Ehira et al., 2009; Busche et al., 2012; Toyoda et al., 2015). Genes regulated by σ^Η^ were analyzed by using the *sigH*-overexpressing *C. glutamicum* R strain and *sigH* deletion strain. In total, 37 genes which were downregulated in the *sigH*-deletion strain and/or upregulated in *sigH*-overexpressing strain were considered to be under the control of σ^Η^ (Ehira et al., 2009). Ten transcriptional start sites (TSSs) were determined by 5’RACE PCR, and the σ^Η^-dependent promoters were localized. Using the sequences of previously determined *C. glutamicum* R σ^Η^-controlled promoters, a consensus sequence was deduced: −35 gGGAAta and −10 ^t^/_c_GTTgaa (Ehira et al., 2009). The set of σ^Η^-dependent promoters was widened in a study of *C. glutamicum* ATCC 13032 in which we used another approach: deletion of the gene encoding the supposed σ^Η^-specific anti-σ factor RshA (Busche et al., 2012). We proved that *rshA* deletion enhanced σ^Η^ activity without influencing stress. In total, 45 σ^Η^-controlled promoters which were found in the two studies were analyzed and the consensus sequence −35 GGAA^T^/_C_ and − 10 GTT was derived (Ehira et al., 2009; Busche et al., 2012). Considering the extent of the σ^Η^ (in *M. tuberculosis* and *C. glutamicum*) and σ^R^ (in *S. coelicolor*) regulatory networks and the variety of their functions, these σ factors can be considered to be global regulators.

Another *M. tuberculosis* ECF sigma factor, σ^E^, forms a regulatory network that has a role in heat, cell surface, and oxidative stress response. It is also essential for various virulence functions (Manganelli et al., 2001; Manganelli and Provvedi, 2010). The σ^E^ regulatory network in *M. tuberculosis* is controlled by a two-component system which involves MprA and MprB transcription factors (Manganelli and Provvedi, 2010).

A large number of genes of the *S. coelicolor* σ^E^ regulon were defined, which were directly driven from experimentally proven or deduced σ^E^-dependent promoters (Tran et al., 2019). Expression of these genes is induced by various antibiotics which affect the cell envelope as a target. More than half of the genes of the regulon encode proteins implicated in cell envelope function. This stress response is coordinated by the two-component systems VanRS and CseBC, in addition to σ^E^ (Tran et al., 2019).

Recently, a basic regulatory network operated by σ factors was described in *Rhodococcus erythropolis*, another member of the Mycolata group, closely related to *C. glutamicum* (Štěpánek et al., 2022). The genes encoding four σ factors (σ^B^, σ^E^, σ^H^, and σ^J^) were found to be most likely transcribed by either RNAP+σ^E^ or σ^H^.

The σ^E^-deficient *C. glutamicum* strain was shown to be sensitive to heat, SDS, EDTA, and lysozyme (Park et al., 2008). Expression of the *sigE* gene is low during the exponential growth phase and increases after heat and cell surface stress (Park et al., 2008) and under growth limitation (Brockmann-Gretza and Kalinowski, 2006). We have shown that the *C. glutamicum* genes *dnaK* (chaperone), *dnaJ2* (chaperone), and *clgR* (transcriptional regulator) are under the control of σ^E^ (Dostálová et al., 2017). In addition, the gene encoding alternative sigma factor σ^Β^, which is involved in responses to various stress conditions, is also regulated by σ^E^ (Šilar et al., 2016). The deduced key sequences of these σ^Ε^-dependent promoters (GGAAC–N_18-19_–GTT) were found to be closely similar to the consensus of *C. glutamicum* σ^Η^-dependent promoters (GGAAT–N_18-19_–GTT; Ehira et al., 2009; Busche et al., 2012) and identical to the consensus of the mycobacterial σ^E^-dependent promoters (Rodrigue et al., 2006). We showed in previous studies that promoters of all these *C. glutamicum* genes are not only recognized by σ^E^, but also by σ^Η^ (Šilar et al., 2016; Dostálová et al., 2017). These results suggested that there is a certain overlap in the promoter recognition specificity of σ^Η^and σ^E^ and that the two corresponding regulons probably overlap to some extent. However, the principal roles of σ^Η^ and σ^E^ appear to be different in *C. glutamicum*: σ^Η^ mainly directs the heat and oxidative stress response, whereas σ^E^ activity is especially associated with the cell envelope stress response (Park et al., 2008). The *sigE* gene is co-transcribed with the σ^E^-specific anti-σ^Ε^ gene *cseE*. The interaction of σ^E^ and CseE was proven by *in vitro* assay (Park et al., 2008). No further study of CseE was performed and the mechanism of σ^E^ release from the inhibition is therefore not known. The protein CseE is homologous to the cognate anti-sigma factor of σ^Η^, named RshA in *C. glutamicum*. Both contain a zinc-associated motif HxxxCxxC that is typical for a class of anti-sigma factors. The structure of this motif was discovered in the crystal structure of the anti-sigma factor RslA in *M. tuberculosis* (Thakur et al., 2010). We may therefore consider the CseE protein as an anti-sigma factor.

We have decided to uncover σ^E^-dependent genes by RNA sequencing analysis of the *cseE* deletion strain, *C. glutamicum* Δ*cseE*. Since several genes were shown to depend both on σ^Ε^ and σ^Η^ (Šilar et al., 2016; Dostálová et al., 2017), we also used σ^Η^ to compare the functions of σ^Ε^ and σ^Η^ using *in vivo* and *in vitro* assays. The main aim was to define the recognition overlap of σ^Η^ and σ^E^ regulons, reveal the differences between the specific promoter sequences and recognition specificities of the two σ factors, and make conclusions about the roles of σ^Ε^ and σ^Η^ in various stress responses.

## Materials and methods

### Bacterial strains, plasmids, oligonucleotides, and growth conditions

*Escherichia coli* DH5α (Hanahan, 1985) was cultivated aerobically in 500-ml flasks containing 70–100 ml of 2xYT medium (Green and Sambrook, 2012) at 37°C. *C. glutamicum* RES167 (Tauch et al., 2002) and *C. glutamicum* RES167Δ*cseE* (named *C. glutamicum* Δ*cseE* here) were cultivated under the same conditions, but at 30°C. The defined chromosomal deletion Δ*cseE* in the *C. glutamicum* chromosome was constructed using the *sacB* gene as a conditionally lethal marker and a double-crossover event as described previously (Busche et al., 2012). The plasmid vectors used are listed in Table 1. The sequences of the oligonucleotides for PCR and DNA cloning are shown in Supplementary Table S1.

**Table 1:** Plasmid vectors used.

Plasmid	Characteristics	Reference
pEPR1	*E. coli-C. glutamicum* promoter-test vector, promoterless *gfpuv* as a reporter, Km^R^	Knoppová et al. (2007)
pEC-XT99A	*E. coli-C. glutamicum* expression vector, IPTG-inducible *trc* promoter, Tc^R^	Kirchner and Tauch (2003)
pRLG770	*E. coli* vector for cloning promoters (templates) for *in vitro* transcription assay, Ap^R^	Ross et al. (1990)
pET2	*E. coli*-*C. glutamicum* promoter-test vector	Vašicová et al. (1998)

### DNA manipulations

DNA isolation, PCR, cutting with restriction enzymes, ligation, and transformation of *E. coli* were done using the standard techniques (Green and Sambrook, 2012). Mutations in *sigH* and *sigE* were constructed with a Q5 Site-Directed Mutagenesis kit (New England BioLabs Inc.).

### Primer extension analysis

Non-radioactive primer extension (PEX) analysis was carried out as described previously (Busche et al., 2012). *C. glutamicum* cells were cultivated in 2xYT medium at 30°C and disrupted with glass beads and a FastPrep FP120 (BIO101) disintegrator. The cell debris was removed by centrifugation, and total RNA was isolated from the extract using a High Pure RNA Isolation Kit (Roche Diagnostics). The primer extension analysis was essentially done using SuperScript III transcriptase (Invitrogen, Carlsbad, CA) with 30 μg RNA and 5 pmol Cy-5-labeled primer CM4 complementary to the vector pET2. The synthesized cDNA was run on PAA gel simultaneously with the DNA sequencing products generated with the same labeled primer in an A.L.F. Sequencer (GE Healthcare, Munich, Germany).

### RNA isolation, cDNA library construction, and sequencing

The whole procedure was done in principle according to the protocols used previously (Albersmeier et al., 2017; Wittchen et al., 2018; Dostálová et al., 2019) with a few modifications. *Corynebacterium glutamicum* RES167 or *C. glutamicum* ∆*cseE* was cultivated in minimal medium CGXII (Keilhauer et al., 1993) supplemented with glucose 2% (w/v) instead of 4% and protocatechuic acid 30 mg/l instead of 0.03 mg/l. The cells were harvested in the exponential growth phase and frozen in liquid nitrogen.

Total RNA was isolated from 3 biological replicates of *C. glutamicum* cells by a Quick-RNA Miniprep Plus kit (Zymo Research). The samples were treated with DNase (Roche Diagnostics) and RNA was purified with an RNA Clean&Concentrator-5 kit (Zymo Research). Ribosomal rRNA was removed with a Ribo-Zero rRNA Removal Kit for bacteria (Illumina). The purity of RNA was then tested with an Agilent RNA Pico 6,000 kit and an Agilent 2,100 Bioanalyzer (Agilent Technologies). TruSeq Stranded mRNA Sample Preparation guide (Illumina) was then used to construct the cDNA library. The constructed cDNA library was then sequenced with Illumina HiSeq 1,500 using a read length of 70 bases.

### Primary 5′-end-specific cDNA library sequencing

A primary 5′-end-specific cDNA library was constructed for sequencing with the aim of defining TSSs using the protocol described previously (Albersmeier et al., 2017; Wittchen et al., 2018) Briefly, rRNA was depleted and the RNA samples were treated with a terminator 5′-phosphate-dependent exonuclease (Illumina) to remove non-primary transcripts.

The primary 5′-triphosphate ends of the primary transcripts were treated with RNA 5′-polyphosphatase to convert them into 5′-mono phosphate ends, and the 5′-adapter was ligated to the produced 5′-ends. Then, reverse transcription with a stem-loop DNA adapter was carried out and the library was amplified. The primary 5′-end cDNA library was then purified and size-selected for fragments approximately 100–1,000 nt in size by gel electrophoresis and quantified. The fragments were finally sequenced with an Illumina MiSeq.

### Read processing, mapping, and identification of transcription start sites

Paired-end reads were mapped to the *C. glutamicum* reference genome sequence accession number BX927147 as described previously (Dostálová et al., 2019). To visualize short read alignments, detect TSSs and analyze differential gene expression, Read Explorer v.2.2 (Hilker et al., 2014, 2016) was used. TSSs were detected as described previously (Wittchen et al., 2018). To detect the real TSSs (+1), the nucleotide was considered to be position +1 if the number of read starts was 10 times higher than at position −1. The false-positives in the candidate dataset of the predicted TSSs that were incorrectly classified automatically were examined and excluded manually.

### Differential gene expression analysis

Differential gene expression analysis of *C. glutamicum* RES167 and Δ*cseE* was carried out with the whole transcriptome data and ReadXplorer v2.2 (Hilker et al., 2016), including the Bioconductor package DESeq2 (Love et al., 2014). The signal intensity value (*a*-value) was calculated by the log2 mean of normalized read counts, and the signal intensity ratio (*m*-value) by log2 fold change. The evaluation of the differential RNA-seq data was done with an adjusted value of p cutoff of *p* ≤ 0.01 and a signal intensity ratio (*m*-value) cutoff of ≥1 or ≤ −1.

Genes with upregulated or downregulated expression in *C. glutamicum* ∆*cseE* were included in the further analysis (FDR < 0.05, M-value>1 for upregulation, M-value<−1 for downregulation).

### *In vitro* transcription

The *in vitro* transcription assay was carried out essentially as described previously (Holátko et al., 2012). The promoter fragments (56–70 nt; with the downstream end at position +3 to +6 relative to TSS) cloned in the vector pRLG770 (Ross et al., 1990) were used as the templates (Dostálová et al., 2017). The holo-RNAP was reconstituted from the RNAP core enzyme isolated from *C. glutamicum* and individual *C. glutamicum* σ factors isolated as His-tagged recombinant proteins from *E. coli* as described previously (Holátko et al., 2012). The RNAP core (100 nM) was mixed with the respective σ factor (σ^A^, σ^B^, σ^E^, or σ^H^) in a molar ratio of 1:15 (σ^A^) or 1:30 (σ^B^, σ^E^, and σ^H^) The holo-RNAP was assembled for 10 min at 37°C. The transcription mixture was incubated for 10 min at 37°C. The transcripts labeled with [α-^32^P]UTP were separated in 5.5% (w/v) polyacrylamide gel. *In vitro* transcription assays were done 2 or 3 times for each promoter, with essentially the same results.

### Promoter activity measurements using a single- and two-plasmid assay

Sigma factors were assigned to individual promoters *in vivo* using the recently described two-plasmid system for *C. glutamicum* (Dostálová et al., 2017). In principle σ factors, which were overproduced from the expression vector pEC-XT99A, initiated transcription from the individual promoters. The activity of these promoters was then measured using the reporter gene *gfp*uv in the promoter-test vector pEPR1 (Dostálová et al., 2017, 2019). The fluorescence of the cell-free extracts was determined with a Safire2 microplate spectrophotometer (Tecan; excitation wavelength, 397 nm; emission wavelength 509 nm). Arbitrary units of the fluorescence intensity of cell extract (AU/mg protein) were normalized to protein concentration, which was determined by the Bradford assay as described previously in detail (Dostálová et al., 2017, 2019). To test the effects of stresses on the activity of promoters, *C. glutamicum* cells carrying pEPR1 with an analyzed promoter were used in a single-plasmid assay. The cells were cultivated in the same way as for the two-plasmid assay in 2xYT medium and when OD_600_ reached 1.0 (time point 0), stress (40°C for 1 h, 0.01% (w/v) SDS and 4% (v/v) ethanol, respectively) was applied. Promoter activity was measured as the fluorescence intensity of the reporter protein GFPuv.

### Homology modeling and molecular dynamics simulations

The homology models of the σ^H^ and σ^E^ domains which recognize the −10 and − 35 sequences of the respective promoters were produced by using the Swiss-Model server (Waterhouse et al., 2018). The crystal structures of *E. coli* σ^E^, PDBid: 4LUP (for −10 element GTC; Campagne et al., 2014) and PDBid: 2H27 (for −35 element GGAAC; Lane and Darst, 2006) were used as templates. The nucleotides within the *E. coli* σ^E^ consensus were replaced to match the consensus for *C. glutamicum* σ^H^ or σ^E^, where necessary. Molecular dynamics simulations were done using the software package AMBER (Salomon-Ferrer et al., 2013) and Linux computer nodes with powerful NVIDIA GPUs that enable the accumulation of 50-ns MD trajectories at 280 K.

## Results

### Transcriptional pattern of the *sigE*-*cseE*-*tatB* operon

**Figure 1:**
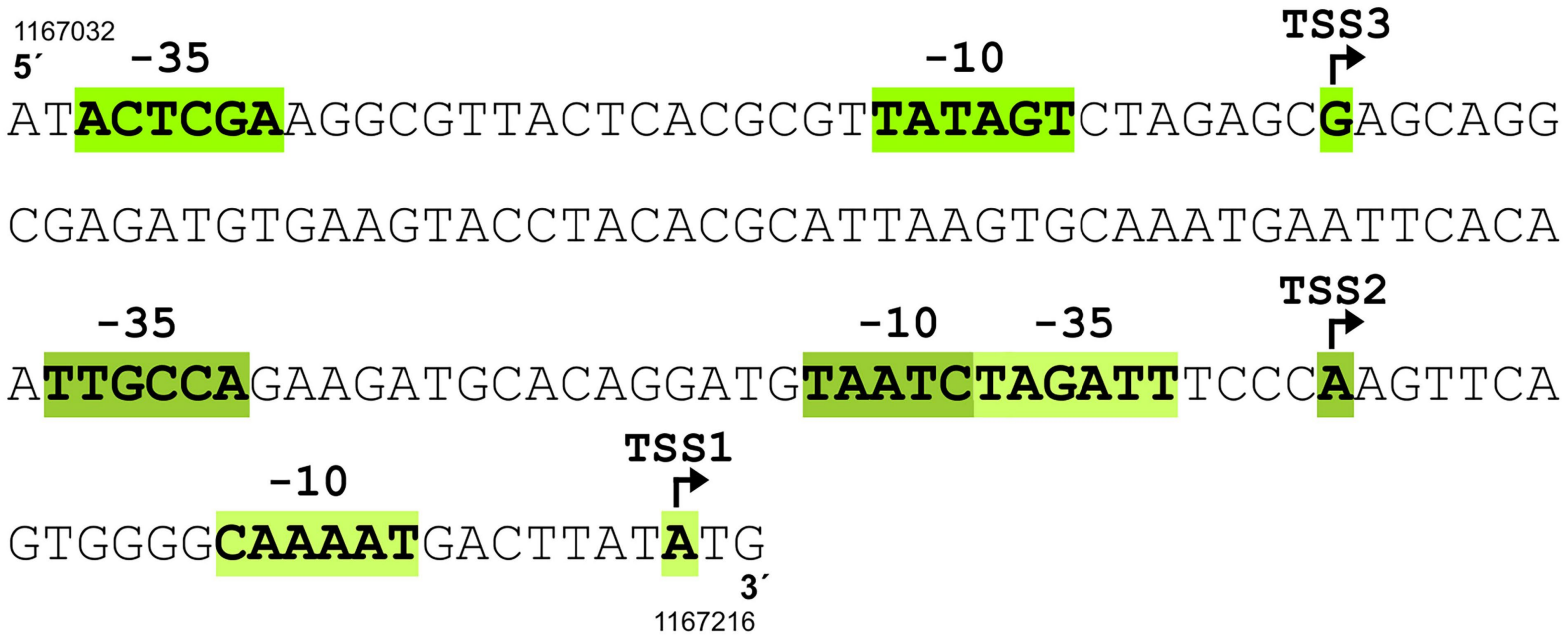
Upstream region of *sigE* gene containing three promoters (P1*sigE*, P2*sigE*, and P3*sigE*) driving transcription from transcriptional start sites TSS1, TSS2, and TSS3. The TSSs were detected by primer extension analysis. The sequences of the corresponding putative −10 elements CAAAAT (P1*sigE*), TAATCT (P2*sigE*), and TATAGT (P3*sigE*) suggest that all three promoters are controlled by σ^Α^. Of the three potential −35 hexamers, only TTGCCA belonging to P2*sigE* conforms to the −35 consensus TTGACA, whereas the other two share little similarity to the consensus. TSS are indicated by bent arrows. Genomic coordinates of the 5′-end and 3′-end of the *C. glutamicum* ATCC 13032 sequence according to GenBank RefSeq BX927147 are shown.

**Figure 2:**
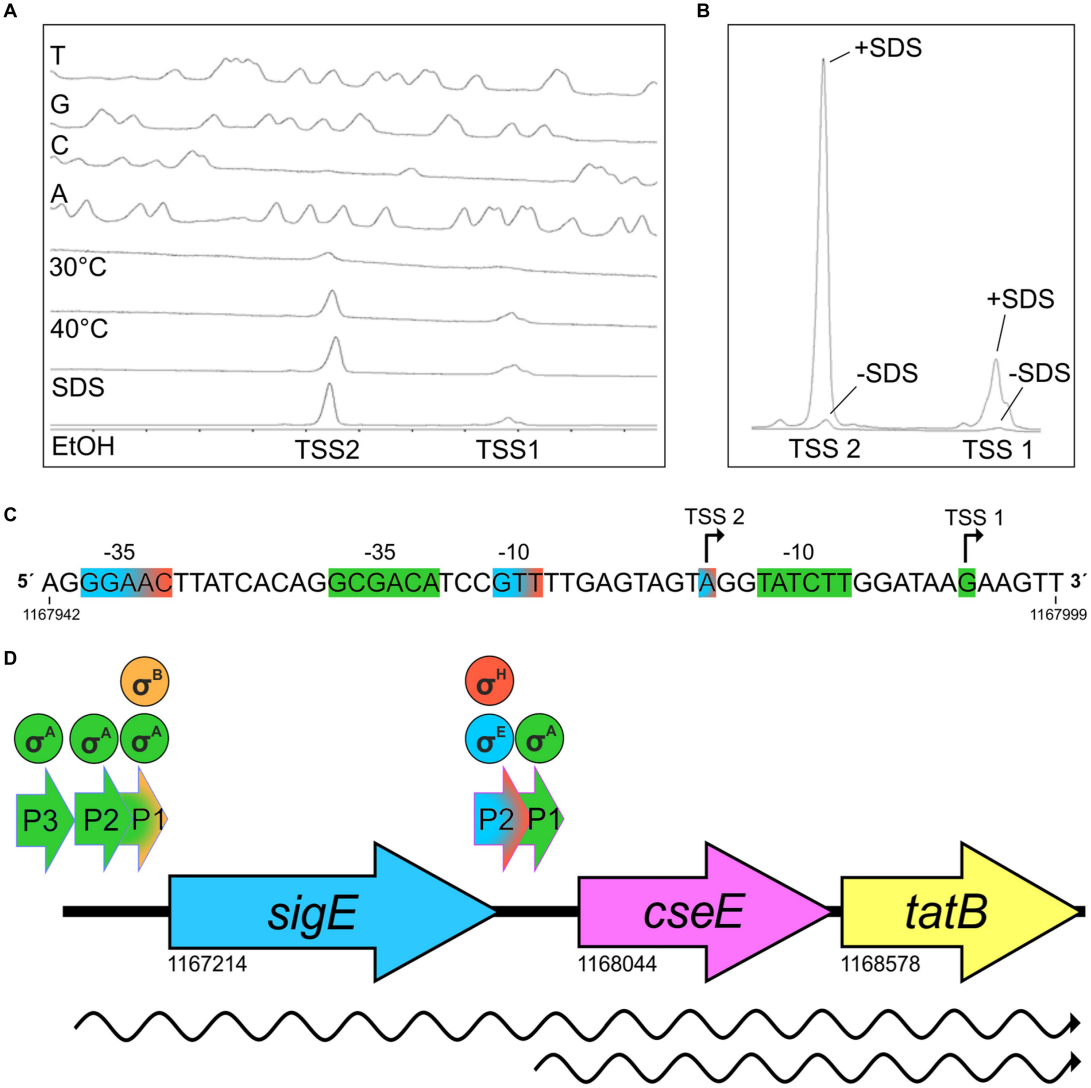
Determination of transcriptional start sites (TSS) of *cseE* gene by primer extension analysis (PEX), sequence of corresponding promoter region, and structure of the whole *sigE*-*cseE*-*tatB* operon. **(A)** Determination of *cseE* transcription start sites by PEX. The bottom peaks represent cDNA synthesized in reverse transcription using RNA from *C. glutamicum* (pET2P*cseE*) cultivated at 30°C or with stresses (40°C for 60 min; 0.01% (w/v) SDS; 4% (v/v) ethanol). The peaks (T, G, C, A) represent the products of sequencing reactions carried out with the same fluorescently labeled primer as that used for reverse transcription. The fluorograms are reversed and labeled complementarily to the respective sequences of the coding strand shown **(C)**. **(B)** The same PEX reaction signals as in 2A (without/with SDS; -SDS/+SDS) shown in absolute size. To compare the strength of the signals, the absolute size of peaks marked -SDS and + SDS are shown. **(C)** Nucleotide sequence of *cseE* upstream region. Transcription start sites (TSS) detected by PEX are indicated by bent arrows. The proposed promoter motifs corresponding to the σ^Α^-controlled TSS1 are highlighted in green, the σ^H^**/**σ^Ε^-controlled TSS2 in blue/red. Genomic coordinates of the 5′ and 3’ends of the promoter sequence taken from GenBank RefSeq BX927147 are shown. **(D)** Scheme of *sigE*-*cseE*-*tatB* operon of *C. glutamicum* and location of promoters and transcripts. The promoters are indicated by short arrows above the operon structure. Transcripts are shown as wavy lines. Sigma factors which are active in transcription from individual promoters are shown in circles above the promoters. The scheme and the genomic coordinates of the initiation codons are based on the *C. glutamicum* ATCC 13032 genome sequence (GenBank RefSeq BX927147) and results of primer extension analyses.

According to the previously published results of RNA sequencing (Albersmeier et al., 2017), the *C. glutamicum* gene encoding the sigma factor σ^E^ (*sigE = cg1271*) forms an operon with the downstream genes *cseE* (*cg1272*, encoding the corresponding anti-σ factor, anti-σ^Ε^) and *tatB* (*cg1273*, twin-arginine translocation pathway protein; gene numbers are according to the genome sequence of *C. glutamicum* GenBank RefSeq BX927147). The sub-operon *cseE*-*tatB* was also detected (Albersmeier et al., 2017). We used primer extension (PEX) analysis to determine the respective *sigE* TSSs. DNA fragments (256 bp upstream of *sigE* and 227 bp upstream of *cseE*) were cloned in the vector pET2 and the constructs were used for PEX assay. Total RNA for PEX was isolated from *C. glutamicum* (pET2 carrying the *sigE* promoter region) and *C. glutamicum* (pET2 with-*cseE* promoter) cells, which were cultivated to the exponential growth phase. Three TSSs were found within the upstream region of *sigE* (data not shown). Two of them, TSS1 at A and TSS3 at G (Figure 1), were identical to the signals obtained by transcriptome sequencing in two studies (Pfeifer-Sancar et al., 2013; Albersmeier et al., 2017). This confirmed the −10 regions of P1*sigE* CAAAAT and P3*sigE* TATAGT. We additionally detected TSS2 at A, which positioned a weaker promoter with the potential −10 element TAATCT an appropriate distance upstream of TSS2. The putative −10 elements CAAAAT (P1*sigE*), TAATCT (P2*sigE*), and TATAGT (P3*sigE*; Figure 1) are similar to the *C. glutamicum* consensus sequence TANAAT of the σ^Α^-dependent (housekeeping) genes (Pátek and Nešvera, 2011; Albersmeier et al., 2017). Nevertheless, no TSS was detected, which might suggest the presence of a σ^Ε^-dependent promoter. We therefore supposed that all 3 promoters are σ^Α^-dependent. The putative −35 regions were not very similar to the already published *C. glutamicum* − 35 consensus sequence TTG^A^/_C_CA (Pátek et al., 2013) or TTGNNN (Albersmeier et al., 2017), but this motif is known to be only weakly conserved in *C. glutamicum* promoters (Pátek et al., 2013; Albersmeier et al., 2017). Upstream of the *cseE* gene, PEX analysis revealed TSS2 at position A (Figures 2A,C), which was identical to the nucleotide found by transcriptome sequencing (Pfeifer-Sancar et al., 2013; Albersmeier et al., 2017). In addition, we detected a weak signal of TSS1 at G, which is 7 nt downstream of the appropriate −10 element TATCTT (Figures 2A,C). The signal at this position has also been previously detected by RNA-seq (Pfeifer-Sancar et al., 2013). The −10 hexamer TATCTT of the promoter P1*cseE* belonging to the weak signal looked again like a typical −10 motif of the σ^Α^-controlled promoters (Figure 2C). The P2*cseE* promoter motifs −35 and − 10, GGAAC–N_18_–GTT (Figure 2C), however, were identical to the sequences of the already described σ^Η^/σ^E^-dependent promoters (Šilar et al., 2016; Dostálová et al., 2017). The strength of PEX signals corresponding to TSS2 promoted by the σ^E^-dependent P2*cseE* was analyzed with RNA isolated from *C. glutamicum* cells afflicted with cell surface stress (addition of SDS). The signal of TSS2 corresponding to the *C. glutamicum* cells cultivated under standard conditions was weak, whereas the respective peak obtained with the cells cultivated with 0.01% (w/v) SDS was very large (Figure 2B). This is in agreement with the assumption that the P2*cseE* promoter is σ^E^-dependent and that the role of σ^E^ is in cell surface stress response. It is noteworthy that the TSS1 peak corresponding to the transcript driven from the housekeeping promoter P1*cseE* was also much larger in response to SDS stress (Figure 2B). This suggests that there is a common regulation of *cseE* transcription from the two promoters, despite them being controlled by different σ factors.

**Figure 3:**
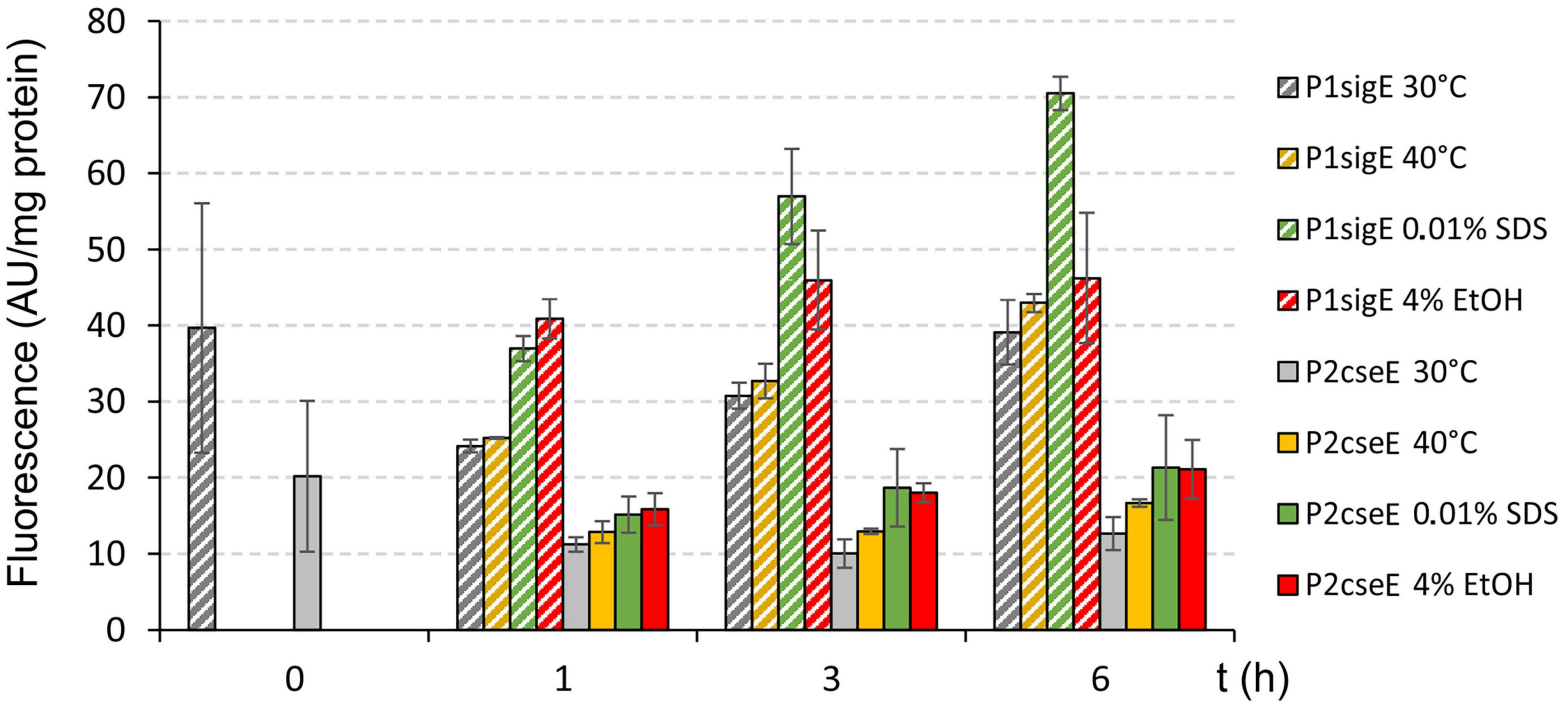
Effects of stresses on activity of P1*sigE* and P2*cseE*. The *C. glutamicum* cells carried the promoter-test vector pEPR1 with the promoter P1*sigE* or P2*cseE*. Promoter activity was measured as the GFPuv fluorescence intensity of cell extracts and is shown as bars representing the respective growth/stress conditions. Error bars depict the standard deviations of three biological replicates.

The effects of the stress conditions on the activities of the main promoters of the operon (P1*sigE* and P2*cseE*) were further examined using a transcriptional fusion of these promoters with the *gfp*uv reporter gene in the promoter-test vector pEPR1 (Figure 3). The strongest effect on P1*sigE* was found with SDS, and a weaker increase in activity was observed with 4% (v/v) ethanol. The effects of these stresses on P2*cseE* activity were only weak and thus not completely convincing. (Figure 3). However, strong effects of 40°C, ethanol (Figure 2A), and especially SDS (Figure 2B) on the P2*cseE* activity were clearly visible in the results of PEX.

**Figure 4:**
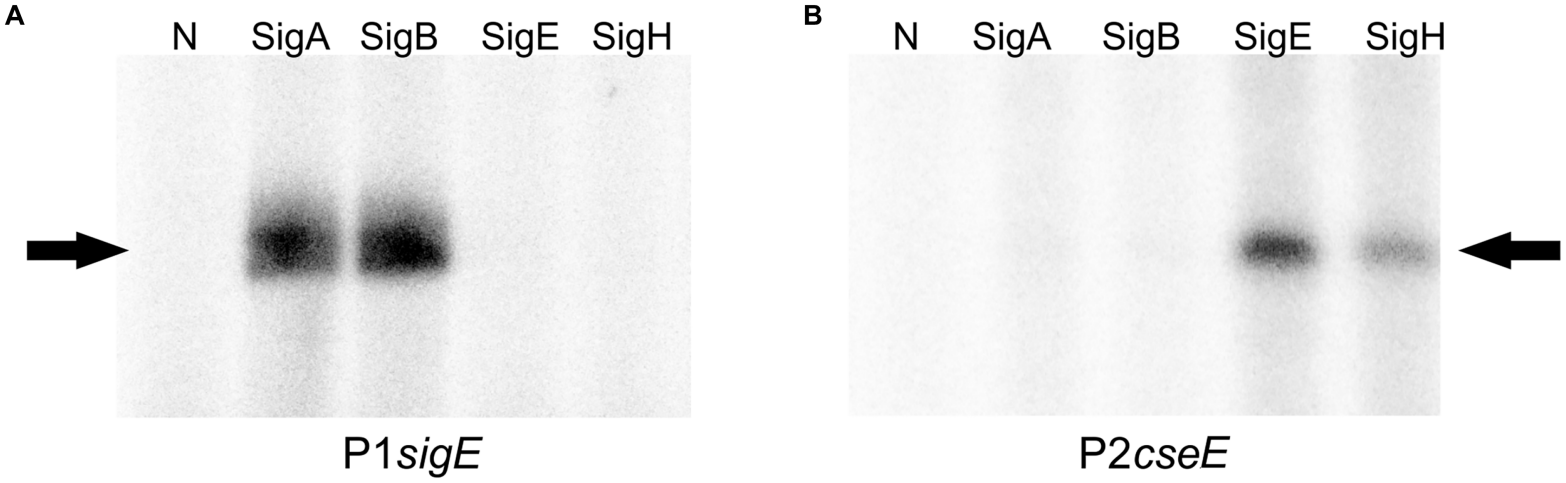
*In vitro* transcription with P1*sigE* **(A)** and P2*cseE* (**B**; the key promoters of the operon) used as templates. Individual sigma factors associated with the *C. glutamicum* RNAP core are shown at the top. N; no sigma factor protein was added to the RNAP core. The specific transcripts are indicated with arrows.

To test the roles of particular σ factors in the recognition of the two strong promoters of the operon by a different method, *in vitro* transcription assay with P1*sigE* and P2*cseE* was carried out using the σ^Α^, σ^Β^, σ^E^, and σ^Η^ factors and the promoters cloned in pRLG770. Signals with approximately the same strength were observed with σ^Α^ and σ^Β^ for P1*sigE*, whereas a strong signal with σ^E^ and a weaker signal with σ^Η^ were detected for P2*cseE* (Figure 4). This result was in agreement with the sequences of the key promoter elements of the tested promoters.

**Figure 5:**
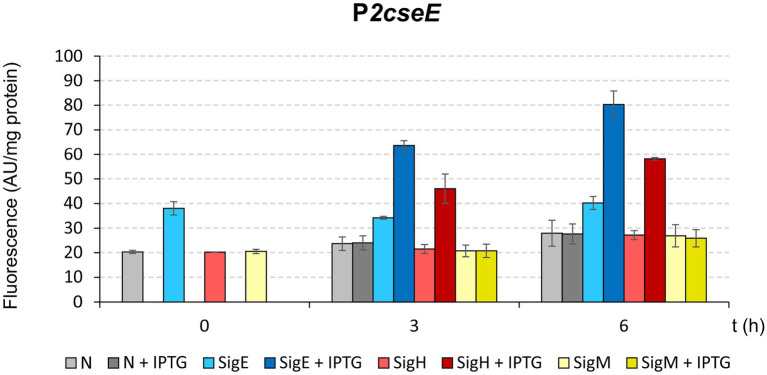
Determination of promoter P2*cseE* activity initiated by *sigE*, *sigH*, or *sigM* overexpression using *in vivo* two-plasmid assay. Promoter activity was measured as GFPuv fluorescence intensity of cell extracts, and is shown as bars representing the respective sigma factors. The *C. glutamicum* strains carried the pEC-XT99A constructs overexpressing *sigE*, *sigH*, or *sigM* after IPTG addition (added at time point 0) and the promoter-test vector pEPR1 carrying the promoterless reporter *gfp*uv gene downstream of the target promoter P2*cseE*. The strains harboring pEPR1 with the P2*cseE* promoter and empty pEC-XT99A (gray bars) or *sigM* (yellow bars) were used as controls. AU, arbitrary units. The standard deviations of three biological replicates are depicted as error bars.

To further test the roles of σ^E^ and σ^Η^ in the transcription from P2*cseE* that was suggested based on the results of *in vitro* transcription (Figure 4), a two-plasmid assay which was developed to prove interaction between σ factors and promoters *in vivo* (Dostálová et al., 2017), was carried out. Expression analysis *in vivo* indicated that P2*cseE* can drive transcription with σ^E^ and a little less also with σ^Η^ (Figure 5). This is in agreement with the result of *in vitro* transcription analysis.

The transcriptional pattern of the *sigE*-*cseE*-*tatB* operon, and the σ factors which control the respective promoters, are summarized in Figure 2D.

### Global transcriptional profiling of the *Corynebacterium glutamicum* Δ*cseE* strain by RNA-seq (differential gene expression analysis)

To view the transcription initiated by σ^E^ at the genome level, we decided to use *C. glutamicum* RES167 and its derivative with a deleted *cseE* gene, respectively, to perform the differential gene expression analysis. Transcriptomes of these two strains were analyzed by RNA-seq. We supposed that σ^E^, which the anti-σ factor CseE could not inhibit in the Δ*cseE* strain, would be active and initiate transcription of the σ^E^-controlled genes even in the absence of a stress stimulus. These conditions are similar to the transcription by σ^H^ in the Δ*rshA* strain. A previous study (Busche et al., 2012) using the *C. glutamicum* Δ*rshA* strain (i.e., deletion of the gene encoding anti-σ^H^) showed that in total 83 genes in 61 transcriptional units were directly or indirectly σ^Η^-dependent.

**Figure 6:**
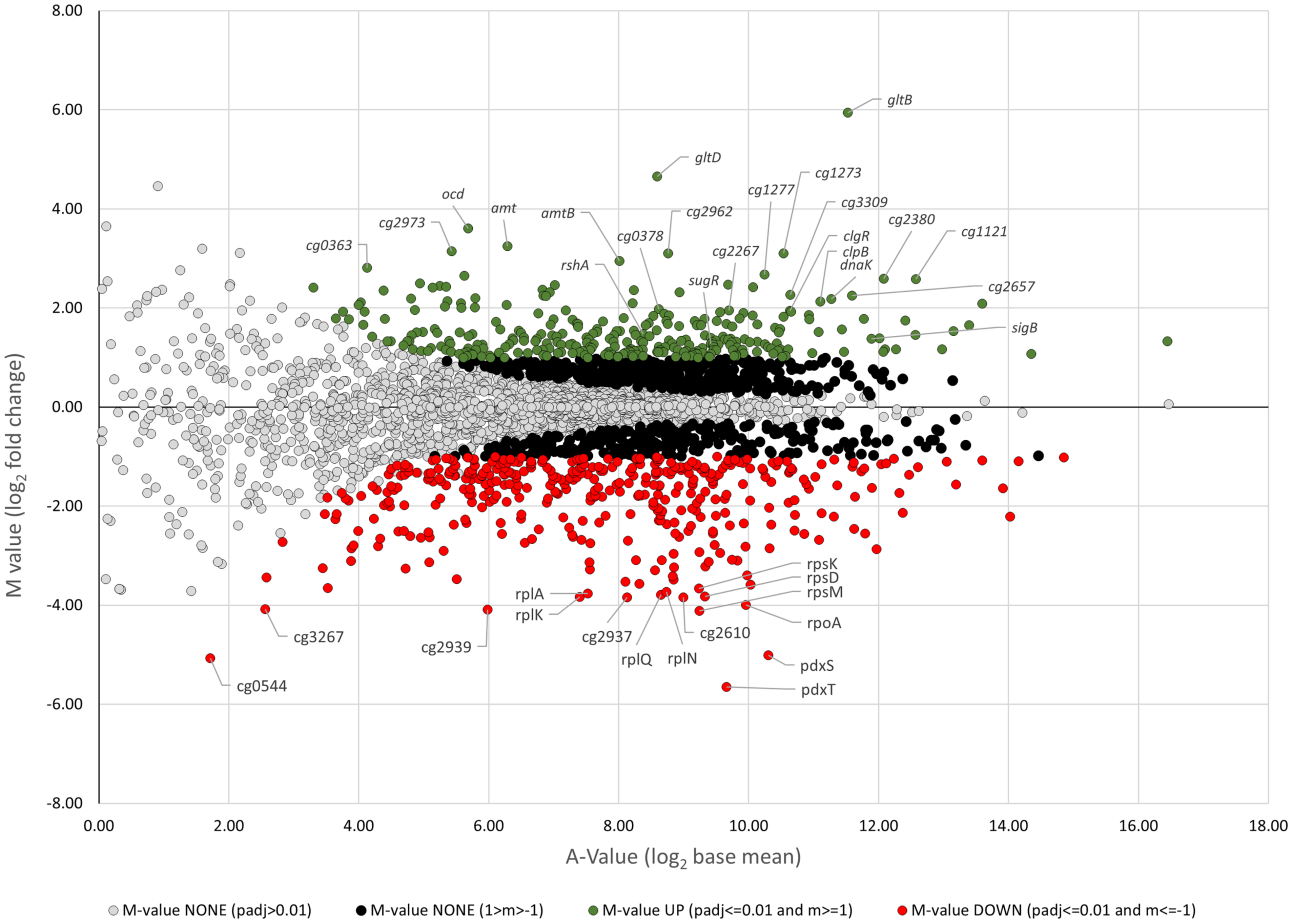
Ratio/intensity (M/A) plot derived from RNA-seq results comparing gene expression of the *C. glutamicum* Δ*cseE* strain with that of the *C. glutamicum* RES167 strain. The data were deduced from three RNA-seq analyses using total RNA probes from three biological replicates. Genes showing significantly increased or decreased transcription levels are marked with green and red circles, respectively. The A value represents the signal intensity, and the M value represents the signal intensity ratio, which corresponds to relative expression changes. The M value cutoff of 1 corresponds to relative expression changes equal to or greater than twofold. Genes were classified as being differentially expressed using the following cut-offs: M-value ≥1.0, upregulation; M-value ≤ −1.0, downregulation.

The ratio/intensity (M/A) plot deduced from RNA-seq results comparing the gene expression of *C. glutamicum* Δ*cseE* with that of *C. glutamicum* RES167 is shown in Figure 6. In total, 694 genes were differentially transcribed (296 upregulated and 398 downregulated) in *C. glutamicum* Δ*cseE* compared to *C. glutamicum* RES167. These genes may be considered as genes whose expression is directly or indirectly modulated by σ^E^, i.e., σ^E^ modulon (Supplementary Tables S2, S3). Our aim was to define the σ^E^ regulon, i.e., the genes under direct control of σ^E^ (these genes are described below, in the section Genes of the σ^Η^/σ^E^ Regulon). We decided therefore to search for the σ^E^-specific promoters upstream of the differentially transcribed genes.

### RNA-seq of the specific 5′-ends of the transcripts resulting in detection of transcription start sites

To localize promoters of the upregulated genes, TSSs were mapped using the results of the sequencing of primary 5′-end-specific cDNA library in the same way as recently with *C. glutamicum* σ^A^-and σ^D^-controlled genes, respectively (Albersmeier et al., 2017; Taniguchi et al., 2017). To detect the actual TSSs (+1), the nucleotide was considered to be position +1 if the number of read starts was 10 times higher than at position −1. Of the upregulated genes (i.e., those which are thought to be members of the σ^Ε^ stimulon), 157 genes were in operons and without TSSs (and therefore without closely located promoters). Upstream of the remaining 139 upregulated genes, 168 TSSs were detected. Some of the genes were transcribed from 2 or 3 or even as much as 4 TSSs. Closely upstream of the detected TSSs, we recognized 150 sequences similar to the consensus sequence of vegetative promoters (i.e., σ^A^-dependent). Using the software Improbizer (Ao et al., 2004), a homogenous group of promoter sequences was detected which were different from the sequences typical for σ^A^-dependent genes in *C. glutamicum*. The sequence GGAAC–N_18-19_–GTT (with a single exception GGAAA–N_19_–GTT in *amtR*), which was identical to the sequences of a few already found σ^Ε^-dependent promoters (Šilar et al., 2016; Dostálová et al., 2017), was recognized in 16 sequences at an appropriate distance upstream of TSSs (Table 2). This group of potentially σ^E^-controlled genes also included the four genes which were previously proved to be transcribed from promoters recognized by both σ^E^ and σ^Η^ (*dnaK*, *dnaJ2*, *clgR* and *sigB*; Šilar et al., 2016; Dostálová et al., 2017). Their putative promoter sequences (approx. 70-bp DNA fragments) were then analyzed and promoter activities were confirmed by *in vivo* and *in vitro* assays. The same procedure for the downregulated genes did not reveal any possible σ^E^-controlled genes and most genes seemed to be σ^A^-controlled (Supplementary Table S3).

**Table 2:** Genes and corresponding promoter sequence motifs −35 and − 10 which were found to be both σ^H^- and σ^Ε^-controlled.

Coding sequence^a^	Gene	−35 and –10^a^	Distance from start codon (nt)	Function
*cg0378*		GGAACA-N_16_-CGTT	92	Putative phage-associated protein
*cg0986* ^b^	*amtR*	GGAAAC-N_17_-CGTT	397	Transcriptional repressor of nitrogen metabolism, TetR family
*cg1121*		GGAACT-N_16_-CGTT	36	Permease, MFS type
*cg1272*	*cseE*	GGAACT-N_16_-CGTT	65	Anti-sigma E factor
*cg1277*		GGAACC-N_16_-CGTT	32	Conserved putative membrane protein
*cg2102*	*sigB*	GGAACT-N_16_-CGTT	25	RNA polymerase sigma factor
*cg2115*	*sugR*	GCAACC-N_16_-CGTT	62	Transcriptional regulator, DeoR-family
*cg2152*	*clgR* (P1*clgR*)	GGAACA-N_16_-AGTT	1	Transcriptional activator of Clp protease genes
*cg2152*	*clgR*	GGAACA-N_16_-CGTT	157	Transcriptional activator of Clp protease genes
	(P2*clgR*)			
*cg2267*		GGAACT-N_16_-CGTT	23	Putative membrane protein
*cg2380*		GGAACA-N_16_-CGTT	41	Putative membrane protein
*cg2515*	*dnaJ2*	GGAACA-N_17_-CGTT	93	Chaperone DnaJ2
*cg2657*		GGAACT-N_16_-CGTT	23	Putative membrane protein, putative pseudogene
*cg3100*	*dnaK*	GGAACA-N_16_-CGTT	120	Chaperone DnaK
*cg3309*		GGAACT-N_16_-CGTT	17	Putative secreted protein
*cg3344* ^c^		GGAACA-N_16_-AGTT	0	3-hydroxypropanoate dehydrogenase, nitroreductase

a All promoters with the exception of cg0986 and cg3344 were discovered by RNA-seq.

b The cg0986 (amtR) gene was proven to be σ^Η^-dependent (Ehira et al., 2009), although there is A at positon − 31 of the promoter in contrast to T at this position in most σ^Η^-specific promoters. See Figure 8.

c The cg3344 gene was proven to be σ^Η^-dependent (Busche et al., 2012), although there is C at positon − 31 of the promoter in contrast to T at this position in most σ^Η^-specific promoters. See Figure 8.

### Activity of the promoters examined by *in vitro* transcription and *in vivo* two-plasmid system

The two techniques, which we developed for the analysis of individual *C. glutamicum* promoters (Dostálová et al., 2017), were applied to confirm the results of the RNA-seq and thus unequivocally assign specific σ factors to the promoters. This combining of the results of two techniques has already provided reliable results with the σ^D^/σ^Η^-dependent promoters (Dostálová et al., 2019). The *C. glutamicum* RES167 cells harboring the expression vector pEC-XT99A with inserted *C. glutamicum sigE* or *sigH* gene and the vector pEPR1 carrying the tested promoter DNA fragment (approx. 70 nt) were used for the *in vivo* two-plasmid assay. The promoter activity was measured as a fluorescence intensity of the GFPuv reporter protein. The chosen promoters were further tested with the *in vitro* transcription system for *C. glutamicum* (Holátko et al., 2012) to confirm the classification of the target promoters.

**Figure 7:**
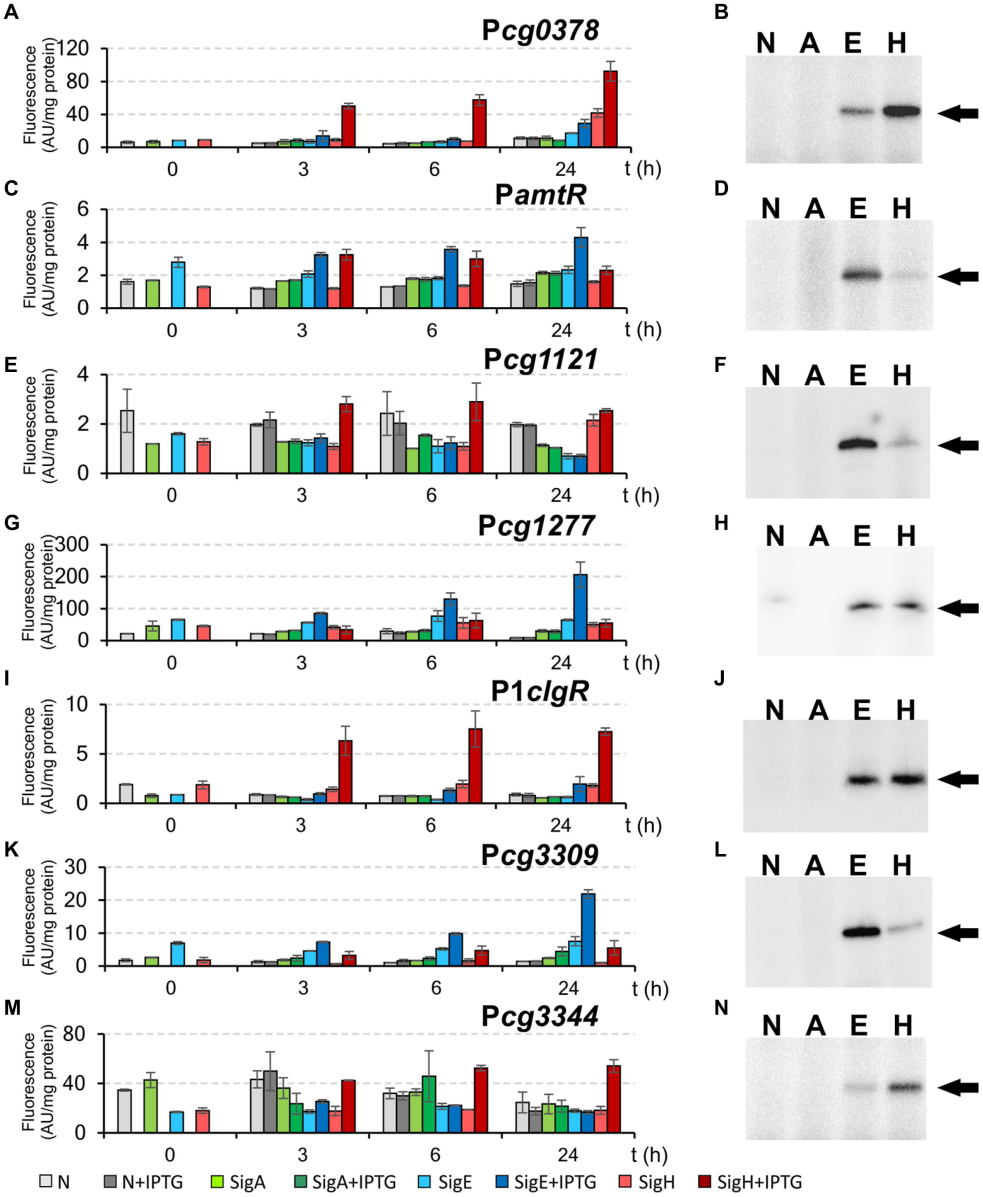
Activity of σ^Η^/ σ^E^-dependent promoters P*cg0378*, P*amtR*, P*cg1121*, P*cg1277*, P1*clgR*, P*cg3309*, and P*cg3344* determined by *in vivo* two-plasmid system **(A,C,E,G,I,K,M)** and *in vitro* transcription **(B,D,F,H,J,L,N)**. The two-plasmid *C. glutamicum* strains carried the pEC-XT99A constructs overexpressing *sigA*, *sigE*, or *sigH* after IPTG addition (at time point 0) and the promoter-test vector pEPR1 carrying the reporter *gfp*uv gene downstream of the tested promoters. The strains carrying pEPR1 with the tested promoters and empty pEC-XT99A were used as the controls (gray bars). The fluorescence intensity of cultures without IPTG is shown as light bars; the fluorescence intensity of cultures with IPTG induction is shown as dark bars. AU, arbitrary units. The standard deviations of three biological replicates are depicted as error bars. Sigma factors (A, E, H; N = no sigma) used for *in vitro* transcription are shown above the autoradiograms (**B,D,F,H,J,L,N)**. The specific transcripts are indicated by arrows. The result of *in vitro* transcription with P1*clgR* shown in panel J (*in vitro* transcription with P1*clgR*) is the same as we published previously (Šilar et al., 2016).

In addition to the promoters, which were mapped by RNA-seq, we selected the promoter P*cg3344*, which was previously found to be controlled by σ^Η^ (Ehira et al., 2009; Busche et al., 2012) and possessed the GGAAC sequence in the −35 region. Moreover, the promoter of the *amtR* (*cg0986*) gene, which was also found to be σ^Η^-dependent (Ehira et al., 2009), and which possessed the atypical sequence GGAAA in the −35 region, was included. The most distinctive examples of σ^Ε^-dependent promoters are shown in Figure 7. The promoters P*amtR* and P*cg3309* were highly active with σ^E^, and weaker activity was also detected with σ^Η^, as measured by both techniques (Figures 7C,D,K,L), whereas the promoter P*cg1277* only exhibited predominant σ^E^-dependent activity *in vivo* (Figures 7G,H). As for P*cg1121*, it was clearly σ^Η^-dependent and very weakly σ^E^-dependent by *in vivo* assay (Figure 7E), whereas an opposite relation was observed in the *in vitro* transcription (Figure 7F). All in all, none of the promoters (including the P2*cseE* promoter of the anti-σ^E^ factor gene, see Figures 4, 5) were found to be exclusively σ^E^-specific in any analysis. Finally, all these σ^E^-dependent promoters were generally active with both σ^E^ and σ^H^ in both *in vivo* and *in vitro* assays (Figure 7). According to the *in vivo* two-plasmid measurements, the most promoters were significantly weaker with overexpressed σ^E^ than σ^H^, whereas the ratio of the strength with RNAP+σ^Η^ and RNAP+σ^E^ in *in vitro* transcription was variable. Interestingly, all the discovered σ^E^-controlled genes (the members of the σ^E^ regulon) thus constitute a gene group which is entirely contained in the σ^Η^ regulon. All σ^E^-dependent promoters (with a single exception of P*amtR*) contained the consensus sequence with the −35 and −10 sequences GGAAC–N_18-19_–GTT.

**Figure 8:**
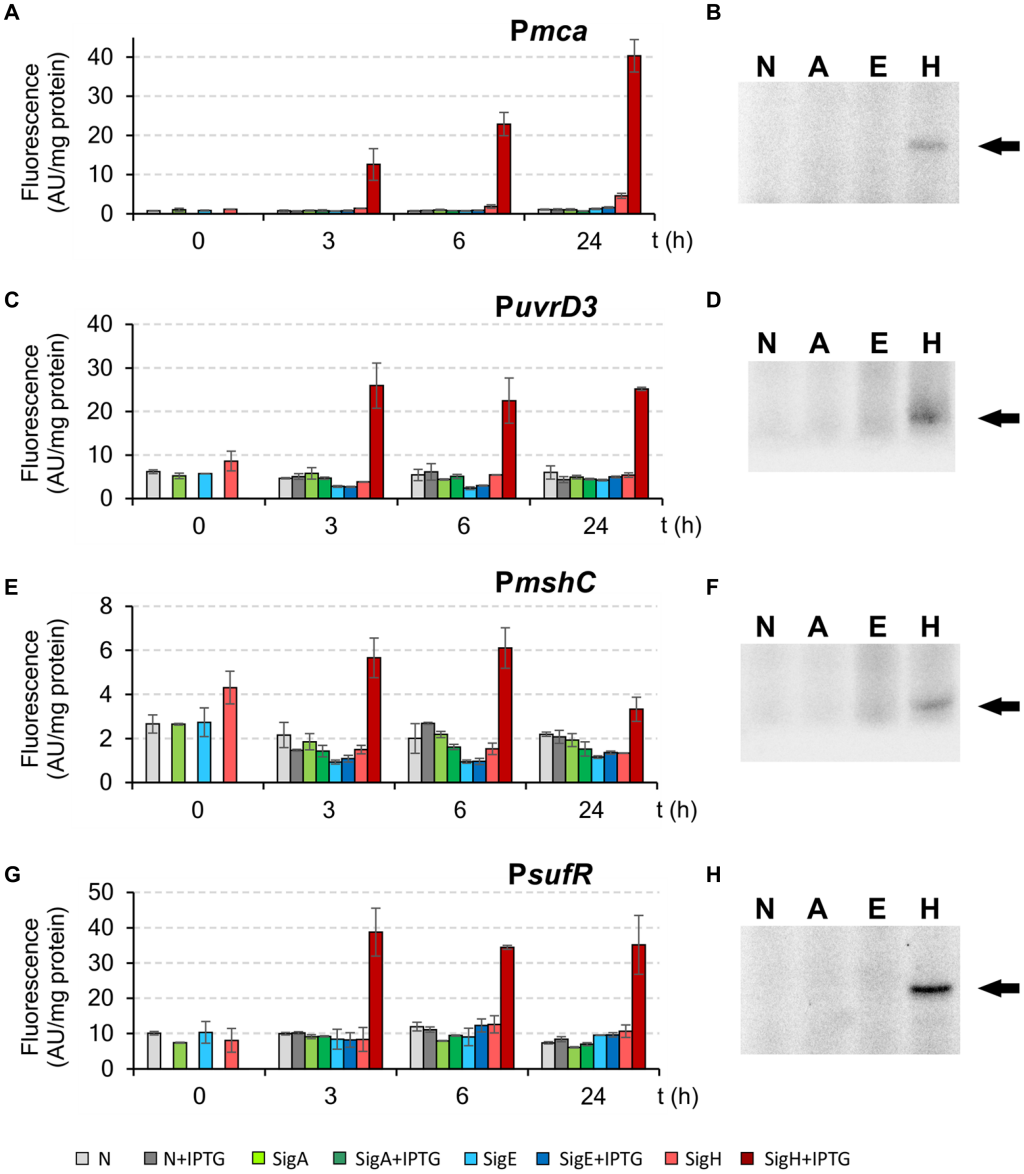
Activity of exclusively σ^Η^-specific promoters P*mca*, P*uvrD3*, P*mshC*, and P*sufR* determined by *in vivo* two-plasmid system **(A,C,E,G)** and *in vitro* transcription **(B,D,F,H)**. The two-plasmid *C. glutamicum* strains carried the pEC-XT99A constructs overexpressing *sigA*, *sigE*, or *sigH* after IPTG addition (at time point 0) and the promoter-test vector pEPR1 carrying the reporter *gfp*uv gene downstream of the tested promoter P*mca*, P*uvrD3*, P*mshC* or P*sufR*. The strains carrying pEPR1 with the tested promoters and empty pEC-XT99A were used as the controls (gray bars). The fluorescence intensity of cultures without IPTG is shown as light bars; the fluorescence intensity of cultures with IPTG induction is shown as dark bars. AU, arbitrary units. The sigma factors (A, E, H; N = no sigma) used for *in vitro* transcription are shown above the autoradiograms **(B,D,F,H)**. The specific transcripts are indicated by arrows. The standard deviations of three biological replicates are depicted as error bars.

As controls, the σ^Η^-dependent promoters P*mca*, P*uvrD3*, P*mshC*, and P*sufR* with the −35 region GGAAT (Table 3) were also used for these assays. Both *in vivo* and *in vitro* techniques clearly confirmed that these 4 tested control promoters are exclusively σ^Η^-specific (Figure 8).

**Table 3:** Exclusively σ^H^-specific genes and corresponding promoter sequence motifs −35 and −10 confirmed by *in vivo* and *in vitro* techniques.

Coding sequence	Gene	−35 and − 10	Distance from start codon (nt)	Function
*cg0877*	*rshA* ^a^	GGAAGA-N_17_-GTT	63	Anti-sigma factor
*cg1127*	*mca*	GGAATG-N_17_-GTT	207	Putative mycothiol S-conjugate amidase
*cg1555*	*uvrD3*	GGAATG-N_17_-GTT	56	DNA/RNA helicase, superfamily I.
*cg1709*	*mshC*	GGAATA-N_17_-GTT	141	L-cysteine, 1D-myo-inositol 2-amino-2-deoxy-alpha-D-glucopyranoside ligase, putative cysteine tRNA synthetase
*cg1765*	*sufR*	GGAATG-N_18_-GTT	30	Predicted transcriptional regulator
*cg3299*	*trxB1* ^b^	GGAATA-N_17_-GTT	33	Thioredoxin

a The results of RNA-seq suggested that the rshA promoter is also σ^Ε^-dependent; however, this was neither confirmed by in vivo nor in vitro assay. The regulation by σ^Ε^ could thus be indirect.

b The trxB1 promoter was analyzed previously (Dostálová et al., 2017).

The comparative analysis of *C. glutamicum* RES167 and *C. glutamicum* Δ*cseE* transcriptomes thus showed that the strategy for detecting the σ^E^-dependent genes using the σ^E^-specific anti-σ factor gene deletion (analogous to previous detection of σ^Η^-dependent genes) was successful. The results confirmed that the *cseE* gene located closely downstream of the *sigE* gene encodes the specific anti-σ^E^ as suggested previously (Park et al., 2008). The results are also consistent with our assumption that σ^E^ would be active in the *cseE* deletion strain that grows under optimal conditions without stress.

### The subtle differences between consensus sequences of σ^Η^- and σ^Η^/σ^E^-dependent promoters

**Figure 9:**
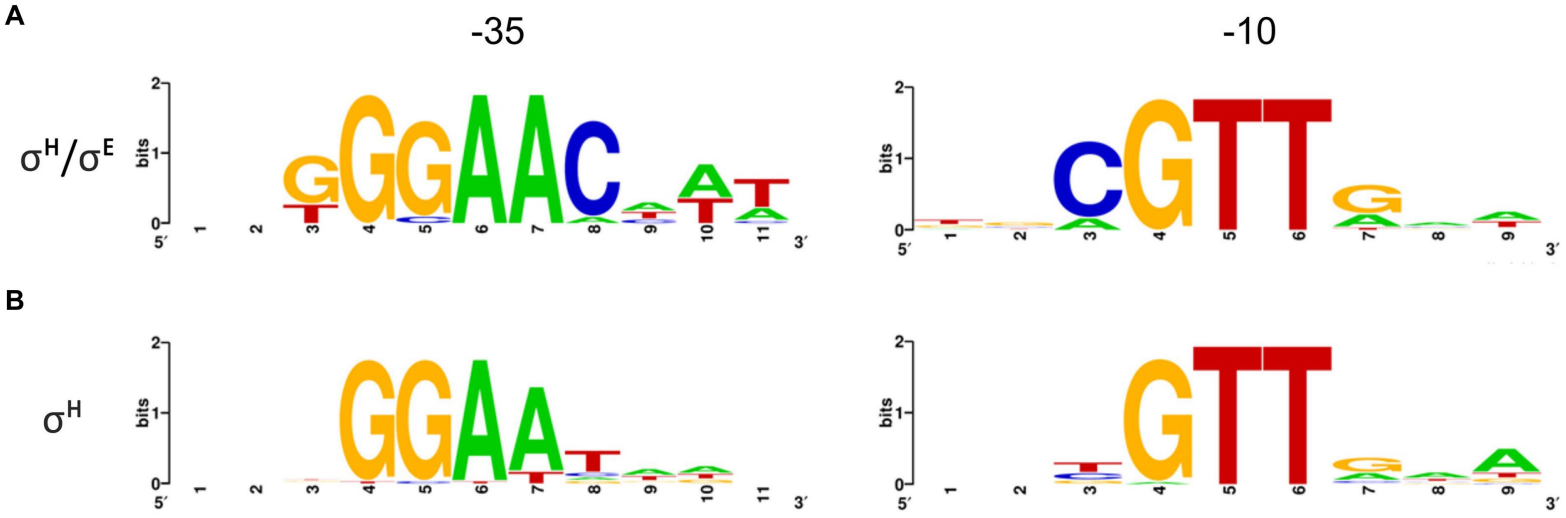
Consensus sequences of σ^Η^/σ^E^-dependent **(A)** and σ^Η^**-**dependent **(B)** promoters defined by Weblogo. Promoters (nt sequences −1 to −50) were aligned at the highly conserved GTT trimer corresponding to the −10 element. σ^Η^/σ^E^ 16 sequences; σ^Η^ 44 sequences. Sequence logos were made with Weblogo3 (Crooks et al., 2004).

The sequences of the 16 σ^Η^/σ^E^-dependent promoters (Table 2) and 44 exclusively σ^Η^-dependent promoters (Ehira et al., 2009; Busche et al., 2012; Toyoda et al., 2015) were separately aligned at the most conserved GTT trimer in the −10 region, and the respective consensus sequences were derived (Figure 9). The consensus sequence of the promoters recognized by σ^Η^/σ^E^ was defined as GGAAC–N_17-18_–cGTT. This consensus differs in a single conserved base (C instead of T) at position −31 from the core consensus GGAAt–N_18-19_–GTT of the σ^Η^-controlled genes (Ehira et al., 2009; Busche et al., 2012). However, RNAP+σ^H^ is also able to initiate transcription from all detected promoters with C_−31_. Moreover, in a few exceptional cases, there is G or A at position −31 in the σ^Η^-controlled promoters. It seems, therefore, that σ^Η^ tolerates any base at position −31, whereas only C at this position ensures transcription with RNAP+σ^E^ (with a single exception of A_−31_ in P*amtR*).

### Homology modeling of σ^H^ and σ^E^ in complexes with −35 elements of σ^H^- and σ^E^-dependent promoters

To see the interactions of the σ subunits with the promoter DNA from another perspective and in atomic detail, we involved computer homology modeling into the analysis. We focused on the difference between the −35 regions of the σ^E^- and σ^H^-dependent promoters in similar way as was done previously for the σ^D^ and σ^H^-dependent promoters (Dostálová et al., 2019).

Recognition of the −35 element of the promoter DNA by stress σ subunits was captured in several X-ray/cryo-electron microscopy structures, which are available in the Protein Data Bank (www.rcsb.org; PDB id: 2H27, 6JBQ, 5ZX2; Lane and Darst, 2006; Guo et al., 2018; Fang et al., 2019). These studies included *E. coli* and *M. tuberculosis* RNAP+σ factors. In fact, all these σ subunits in the published models have the same fold. Moreover, their positioning relative to the −35 element of promoter DNA is also the same in all of them.

The 2H27 structure for σ^E^ from *E. coli* (Lane and Darst, 2006) was used as a template for creating homology models for *C. glutamicum* σ^E^ and σ^H^. The non-template/template DNA strand in the 2H27 crystal structure has the GGAACTT/CCTTGAA sequence, which was in fact identical to the sequence of the *C. glutamicum* P*cg3309* promoter in the key region −35 to −29. Therefore, we used P*cg3309* as an example of a predominantly σ^E^-dependent promoter. P*trxB1* (GGAATAA/CCTTATT) was taken as a typical strong exclusively σ^Η^-specific promoter.

**Figure 10:**
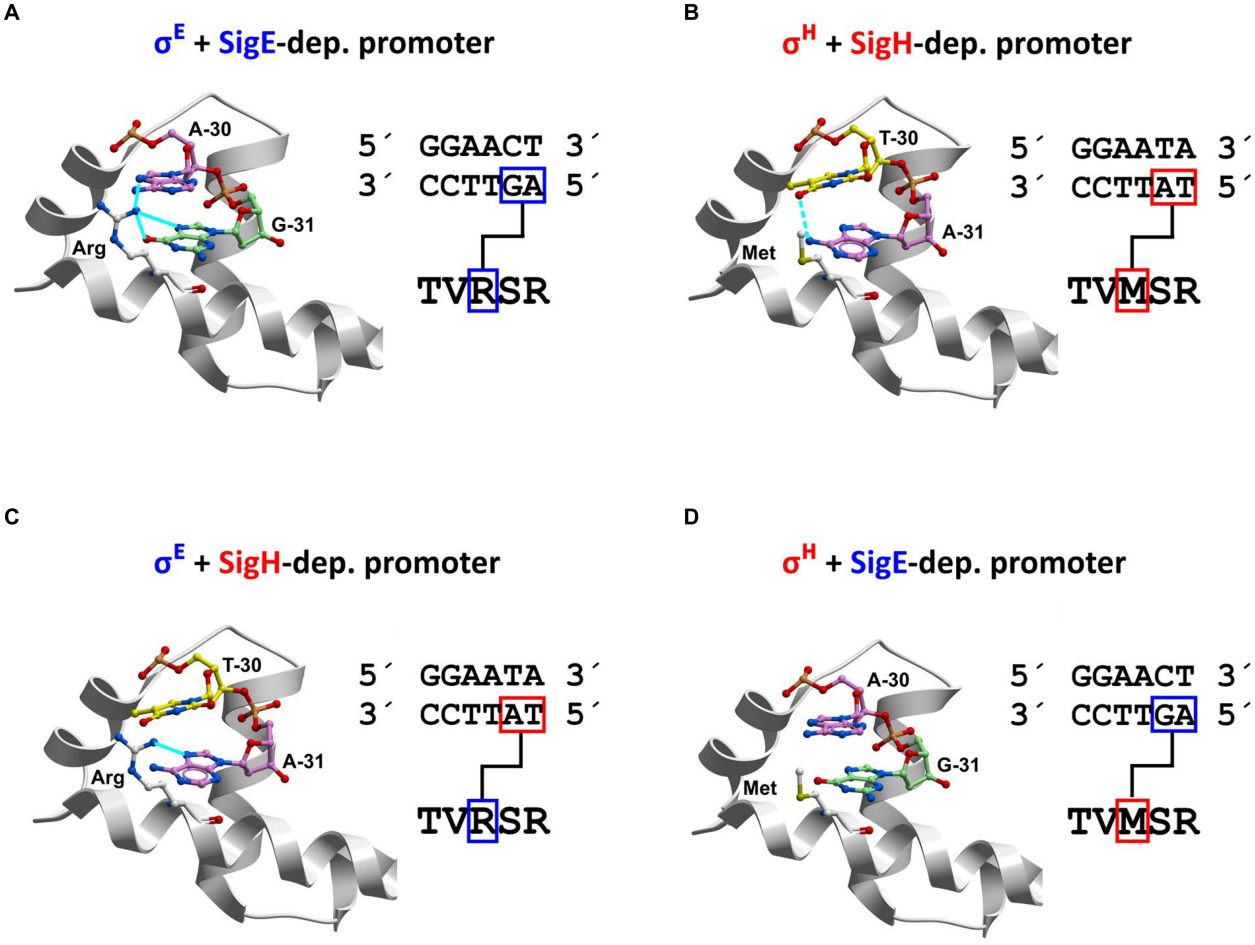
Recognition of nucleotides at promoter positions −31 and − 30 within non-coding (template) strand by σ^E^ and σ^H^ (homology modeling). The −35 sequences of the predominantly σ^E^-dependent promoter P*cg3309* and exclusively σ^Η^-dependent promoter P*trxB1* are shown. Four unique combinations of the key interacting partners (amino acids R/M and dinucleotides at positions −31 G/A, −30 A/T) can occur (i.e., R-GA, M-AT, R-AT, M-GA). DNA: red = oxygen; blue = nitrogen; A: purple = carbon; T: yellow = carbon; G: green = carbon. Amino acids: white = carbon; blue = nitrogen; yellow = sulfur; red = oxygen. Interactions: **(A)** Arg-GA; **(B**) Met-AT; **(C)** Arg-AT; **(D)** Met-GA. Arginine R has a positively charged side chain, which forms stabilizing salt bridges with negatively charged phosphate groups of nucleic acids. In our models, the arginine side chain can form stabilizing hydrogen bonds with neutral nucleic acid bases G_−31_ and A_−30_ in the complex of σ^E^ with predominantly σ^E^-dependent promoters (P*cg1121*, P*cg3309*, P*sigB* or P*cseE*; Figure 11A). In contrast, if the hydrophilic side chain of R is pushed into the immediate vicinity of the hydrophobic methyl group of T_−30_ (as in the case of σ^E^ and P*trxB1*; Figure 11C), it will prevent hydrophilic R from coming into energetically advantageous contact with water molecules. Therefore, the close interaction of R with the T_−30_ base can destabilize complexes of σ subunits and promoters (Figure 11C). Methionine (M) is a hydrophobic amino acid that does not form hydrogen bonds with nucleic acid bases. From the point of view of the overall energy of the system, it is advantageous if the hydrophobic motifs come together, which allows the surrounding water molecules to form the maximum number of hydrogen bonds. Therefore, the stabilizing interactions can occur upon contact of M with the hydrophobic methyl group of T_−30_ of the template strand (in P*trxB1*, P*rshA*, and P*mshC*; Figure 11B). If the hydrophobic side chain of methionine comes close to G_−31_ A_−30_ as in P*cg3309*, neither hydrogen bonds nor hydrophobic interaction can be formed and a weak coupling results (Figure 11D).

Our homology models showed that the side chains of arginine R185 (in σ^E^) or methionine M170 (in σ^H^) interact with nucleotide bases (within the non-coding strand) at positions −31 and − 30 of the promoters (Figures 10A,B).

### Design of σ^Ε^ and σ^Η^ mutagenesis and testing the effects of mutant sigma factors on activities of the σ^H^ or σ^H^/σ^E^-controlled promoters

Based on the conclusions from homology modeling, mutations in the key amino acids arginine R185 (in σ^E^) or methionine M170 (in σ^H^; i.e., R185 → M185 in σ^E^, and M170 → R170 in σ^H^) were proposed. The wild-type σ^H^, σ^E^, and the modified sigma factors σ^H^mut and σ^E^mut can form with P*cg3309*, P*trxB1*, and P*cseE* in total four unique combinations of key interactions between amino acids R or M and dinucleotides AT and GA at positions −31 and − 30 in the template strand, i.e., R-GA, M-AT, R-AT, M-GA (Figure 10). In addition, there are the combinations M-CT and R-CT that occur with P*rshA*, which are analogous to M-AT and R-AT. In these combinations, the amino-group of cytosine may interact in a similar way to the amino group of adenine.

**Figure 11:**
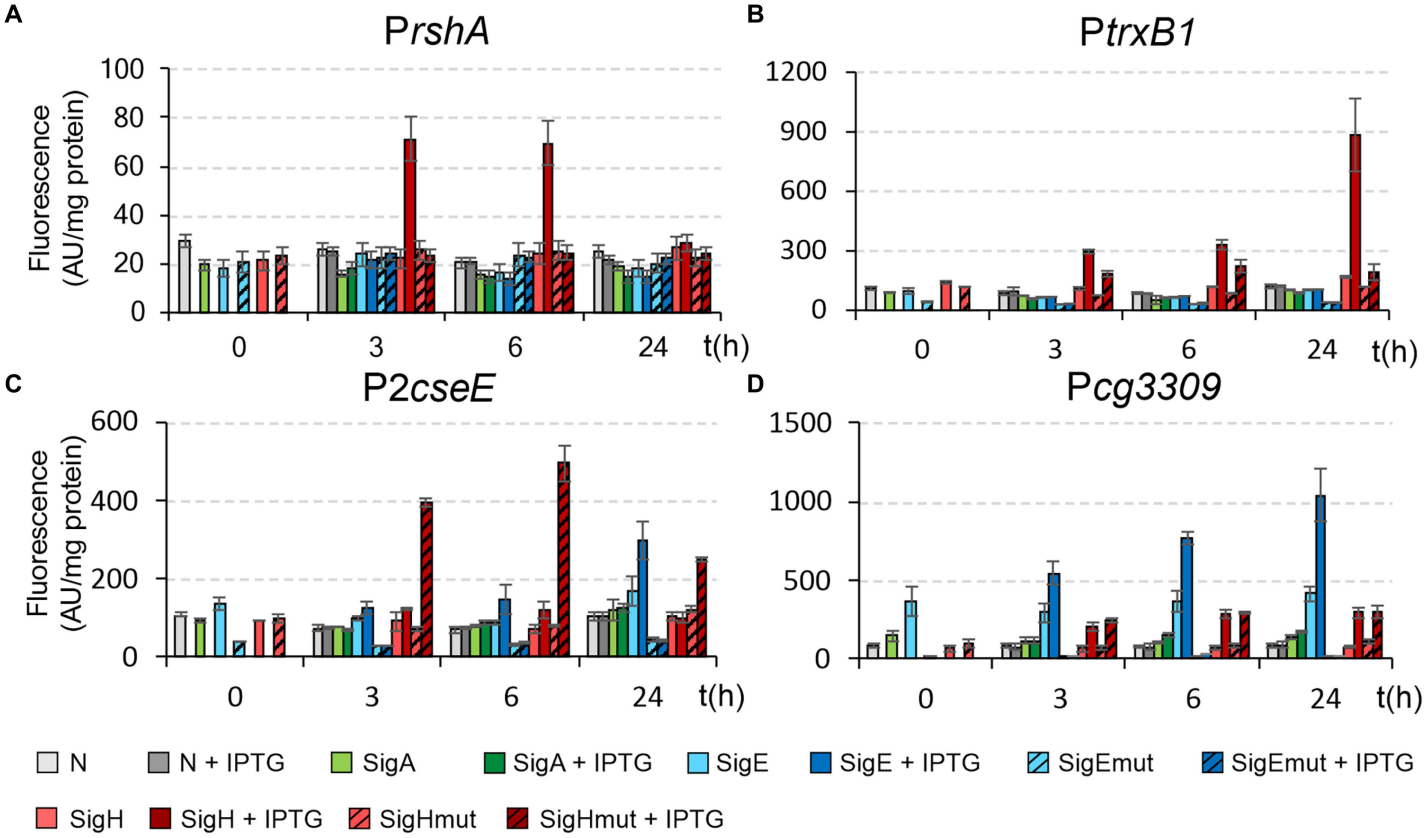
Activity of σ^H^-controlled promoters P*rshA* **(A)** and P*trxB1* **(B)** and σ^H^/σ^E^-controlled promoters P2*cseE* **(C)** and P*cg3309* **(D)** with wild-type and mutant σ^H^ and σ^E^. The activities were determined by two-plasmid assay. The *C. glutamicum* strains carried the pEC-XT99A constructs overexpressing *sigA*, *sigE*, *sigE*mut, *sigH*, or *sigE*mut after IPTG addition (at time point 0) and the promoter-test vector pEPR1 carrying the reporter *gfp*uv gene downstream of the tested promoters. The strains carrying pEPR1 with the tested promoters and empty pEC-XT99A were used as the controls (gray bars). The fluorescence intensity of cultures without IPTG is shown as light bars; the fluorescence intensity of cultures with IPTG induction is shown as dark bars. AU, arbitrary units. The standard deviations of three biological replicates are depicted as error bars.

The genes encoding the mutant σ factors were constructed and cloned into the expression vector pEC-XT99A. The plasmid constructs of pEC-XT99A with the genes encoding σ^H^ (M170), σ^E^ (R185), σ^H^mut (R170), and σ^E^mut (M185) and constructs of pEPR1 with the selected promoters were used for creating combinations of the two plasmids (pEC-XT99A + *sig* and pEPR1 + promoter) in *C. glutamicum* clones. In addition to the predominantly σ^E^-controlled P*cseE* and P*cg3309* promoters and exclusively σ^H^-dependent P*trxB1* promoter, we decided to also test P*rshA*. This promoter is one of a few exceptions among the strong σ^H^-dependent promoters with G at position −31 (instead of T) and it can be thus supposed that M170 in σ^H^ would interact with −31 and − 30 CT (instead of AT) in the template strand. The promoter activities in the resulting clones were measured by the standard *in vivo* procedure as the intensity of fluorescence produced by GFPuv (Figure 11).

The use of σ^Ε^mut and σ^Η^mut enabled us to evaluate the results of promoter activity measurements (Figure 11) in connection with the hypotheses based on homology modeling (Figure 10).

The activity of the predominantly σ^E^-dependent promoters P*cseE* and P*cg3309* vanished completely when the σ^E^mut was used (Figures 11C,D). Apparently, their activity depends strictly on R185 in σ^E^. We expected that the activity of these promoters, which are to a lower extent also active with σ^H^, will be higher with σ^H^mut carrying R170. This is true with P*cseE*, whereas the activity of P*cg3309* was approximately the same with σ^H^ and σ^H^mut (Figures 11C,D). Apparently, sequences outside of the conserved motifs −35 and −10 also play significant roles in the activity of the individual promoters. As for the exclusively σ^H^-dependent P*trxB1* and P*rshA*, the mutation in σ^H^mut (M170 → R170) also impaired their activity: to a large extent for P*trxB1* and completely for P*rshA* (Figures 11A,B). Neither of these strictly σ^Η^-specific promoters were recognized by σ^E^mut. Although σ^E^mut carried methionine occurring in σ^Η^ in the key position, this single alteration in σ^E^ did not suffice for the recognition of these σ^Η^-specific promoters.

We can conclude that the homology models of σ^E^- and σ^H^ interactions with the respective promoters were generally confirmed, although differences in promoter sequences outside the −10 and − 35 elements can significantly modulate the promoter activity.

### Genes of the σ^H^/σ^E^ regulon

Genome-wide differential gene expression analysis by RNA-seq detected 296 upregulated genes using the *C. glutamicum* Δ*cseE* strain. This group forms the σ^E^ stimulon, in which genes directly or indirectly dependent on σ^E^ activity can be identified. However, only 15 genes were strictly defined as members of the σ^E^ regulon based on the presence of the σ^E^-dependent promoters (*clgR* is transcribed from 2 σ^E^-dependent promoters). Most of these genes (Table 2) are apparently related to maintaining cell envelope integrity or to protein quality control. The *cseE* gene encoding the cognate anti-σ is a natural part of the regulatory circuit, which controls the σ^E^ function (Park et al., 2008). The *sigB* gene, which was previously found to be under control of a σ^Η^/σ^E^-dependent promoter (Dostálová et al., 2017), encodes the σ factor, which plays a key role in the activation of cell functions essential for *C. glutamicum* cell survival during the transition and stationary growth phases and in response to various stresses (Larisch et al., 2007). Among them, the effects of acids, ethanol, cold, and heat shock may finally lead to the cell envelope stress response. Thus, the *sigB* gene is a member of the σ^Η^/σ^E^ regulon.

It was shown that the presence of misfolded proteins in the cell envelope activates σ^E^ in some bacteria. In agreement with this, the main general function of chaperones such as DnaK and DnaJ2, which are encoded by the σ^H^/σ^E^ -activated genes *dnaK* and *dnaJ2* in *C. glutamicum*, is to promote proper protein folding and prevent aggregation of the incompletely folded proteins.

Another essential task in protein quality control is removing proteins which have been irreversibly damaged by stresses. This function is fulfilled by Clp proteases. The transcription of the gene *clgR* that encodes a regulatory protein which activates the expression of these proteases in *C. glutamicum*, was found to be induced by the heat shock response mediated by σ^H^ (Engels et al., 2004). In mycobacteria, *clgR* is also induced by oxidative and detergent stress. These responses are activated by σ^H^ and σ^E^, respectively (Manganelli et al., 2002).

SugR regulator is a transcriptional repressor of the central carbon metabolism of *C. glutamicum*. Repression of the sugar metabolism may also be connected to the slowed growth during the stress responses.

The function of the *cg3344* gene encoding 3-hydroxypropanoate dehydrogenase is related to energy production and conversion. This enzyme is involved in the pathway of pyrimidine degradation which may be connected to the slowed growth rate of the cell culture and energy saving during the defense reaction to stress effects.

The *cg1121* gene encodes permease of the MFS type. The product belongs to the large family of transporters (efflux pumps) which confer bacteria resistance to various compounds, e.g., antibiotics. The *tatB* gene (twin-arginine translocation pathway protein) which is in the operon with *sigE* and *cseE* is most likely also connected to the stress response functions in *C. glutamicum*. The Tat proteins generally have a role in translocating fully folded proteins from the cytoplasm across the cytoplasmic membrane. Among them, redox proteins may be connected to the oxidative stress response (Montero et al., 2019). In *E. coli*, N-acetylmuramoyl-L-alanine amidase, which is transported by the Tat protein system, acts on the peptidoglycan structure, and may thus be involved at some stage in envelope stress response. Recently, SDS as a cell envelope stressor was found to induce expression of the *tatABC* operon in *Salmonella* (Rogers et al., 2022). The same Tat system was also observed to counteract severe oxidative stress and starvation when *Bacillus subtilis* grows in a NaCl-depleted medium (Prajapati et al., 2021).

Four other genes encode putative membrane proteins which may also be related to cell envelope functions. In conclusion, nearly all uncovered members of the *C. glutamicum* σ^Η^/σ^Ε^regulon have some function related to the cell envelope and/or various stresses.

## Discussion

Sigma E is a major regulator in the cell envelope stress response in various Actinobacteria. In *M. tuberculosis*, σ^E^ also controls heat and oxidative stress response (Manganelli and Provvedi, 2010), whereas it coordinates stress response to various antibiotics in *S. coelicolor* (Tran et al., 2019). In addition to cell surface stress response, σ^E^ plays a role in responses to heat stress and slow growth under nutrient-limiting conditions and the stationary phase in *C. glutamicum* (Park et al., 2008; Pátek and Nešvera, 2011). It seems, therefore, that σ^E^ together with σ^H^, which is involved in heat and oxidative stress response and DNA repair in Actinobacteria (Ehira et al., 2009; Busche et al., 2012; Sharp et al., 2016; Park et al., 2019), fulfill major functions in the defense of the bacterial cells against the main threats to their integrity. Moreover, both RNAP+σ^E^ and σ^H^ initiate the transcription of *sigB* in *M. tuberculosis* (Raman et al., 2001), *C. glutamicum* (Dostálová et al., 2017) and *R. erythropolis* (Štěpánek et al., 2022). The primary-like σ^B^ factor of *C. glutamicum* activates the genes in response to salt, ethanol, acid, cold and heat stresses (Halgasova et al., 2002; Larisch et al., 2007; Barreiro et al., 2013) and under oxygen deprivation (Ehira et al., 2008). Generally, σ^B^ is highly active during the transition phase between the exponential and stationary growth phases in *C. glutamicum* (Larisch et al., 2007). RNAP+σ^B^ most likely also contributes to the transcription of *sigE* and *sigH* genes. The intricate regulatory network of these three σ factors integrates signals from various stress conditions and enables cells to overcome unfavorable conditions *via* highly coordinated physiological responses.

In some bacteria (e.g., *E. coli, Salmonella*, and *Burkholderia cenocepacia*), an important role in controlling nitrogen metabolism is played by alternative sigma factor σ^54^, which is structurally and functionally different from the σ^70^-type sigma factors (Lardi et al., 2015). Such an σ^54^ factor is missing in *C. glutamicum*. We found that the gene encoding the key regulator of nitrogen metabolism AmtR was under the control of σ^E^ (and partially σ^H^), although it was not detected as upregulated according to the RNA-seq results. Moreover, many genes of the AmtR regulon (Beckers et al., 2005), which are involved in nitrogen metabolism (*amt, amtB, crnT, cg1918, urtA, urtB, urtC, urtE, glnK, codA, ureA, ureB, ocd*, and *soxA*) were induced in *C. glutamicum* Δ*cseE*, but they were not found to be directly under an σ^E^-dependent promoter. We can speculate that σ^E^ and σ^H^ together with AmtR (and probably some other transcriptional regulators) form a functional module which is responsible for regulating the uptake and assimilation of nitrogen sources.

One of the main aims of this study was to find differences between the σ^Η^- and σ^Ε^-dependent promoters since the class of the promoters upstream of the genes defines a regulon. Surprisingly, we found that the activities of the two σ factors were substantially overlapping and that there is a specific group of σ^Η^/σ^Ε^-dependent promoters. The difference of the two promoter classes (σ^Η^- and σ^Η^/σ^Ε^-dependent) was found mainly in the −35 region of the consensus sequence: σ^E^ could only recognize promoters with the −35 GGAAC sequence, whereas σ^Η^ recognized −35 GGAAN, although −35 GGAAT was the most frequent.

In *M. tuberculosis*, similar features were found (although based on less promoter sequences): C at position −31 was essential for recognition by σ^Ε^, whereas any base at −31 allowed recognition by σ^Η^, although T prevailed (Song et al., 2008). However, a specific class of σ^Η^/σ^Ε^-dependent promoters was not described in *M. tuberculosis*, probably due to lower number of promoters analyzed and different methods used. Interestingly, all four analyzed σ^Η^-dependent promoters showed the −35 GGAAC sequence in *R. erythropolis* (Štěpánek et al., 2022) in our recent study. Two of them seemed to be σ^Η^/σ^Ε^-dependent. The results support the idea that the promoter sequences of promoters recognized by σ^Η^, σ^Ε^, *and* σ^D^ (Dostálová et al., 2019) in *C. glutamicum* (and probably also in the closely related Actinobacteria) and the corresponding σ regulons to various extent overlap. These similarities in structure and activity are most probably based on similar sequences of the key amino acids in σ factors.

The genes encoding stress σ factors in various Actinobacteria, such as *Mycobacterium* (Donà et al., 2008), *Rhodococcus* (Štěpánek et al., 2022), and *S. coelicolor* (Paget et al., 1998), are mostly autoregulated, i.e., they are transcribed by RNAP holoenzymes containing the σ factors which they encode. In contrast, we found that the *C. glutamicum sigE* gene is expressed from three σ^A^- and/or σ^B^-dependent promoters. Instead of the σ^E^-encoding gene, the gene *cseE* encoding the anti-σ^Ε^factor was found to be driven from the σ^H^/σ^E^ -dependent promoter. To compare the transcriptional organization of the *C. glutamicum sigE*-*cseE* operon with that of other *Corynebacterium* species, we analyzed the respective sequences (deposited in KEGG database) of 82 *Corynebacterium* species. A stress promoter with the consensus motifs (GGAAC–N_18-20_–GTT) was only found upstream of the *sigE* gene of 5 *Corynebacterium* species. The autoregulation of *sigE* is thus apparently rare in corynebacteria. Further, a stress promoter with the same structure was found upstream of *cseE* in 11 *Corynebacterium* species. In conclusion, both the *sigE* and *cseE* genes are mostly transcribed from vegetative promoters in the majority of the corynebacteria. In four species (*C. imitans, C. aquilae, C. sphenisci, and C. ureicelerivorans*) the *cseE* gene is clearly missing. The strategy for the control of *sigE* expression thus developed divergently in corynebacteria.

*C. glutamicum cseE* is transcribed from two promoters, σ^Α^-dependent P1*cseE* and σ^Η^/σ^Ε^-dependent P2*cseE* (Figure 2). Primer extension analysis (Figure 2C) showed that both signals representing the two TSSs were enlarged in presence of SDS. The co-regulated transcription from the two promoters may be due to a still unknown transcriptional regulator which binds close to *cseE* promoter region. Transcription of many stress-responsive genes was found to be controlled by such regulators rather than by alternative σ factors (Hünnefeld et al., 2019). Some stress promoters are regulated by both DNA-binding transcription factors and RNA polymerase-binding sigma factors. Then, the two close promoters may be co-operatively regulated by transcription factor although they are transcribed with different sigma factors. We suppose that such situation may occur for example at σ^Α^-dependent P1*dnaK* and σ^Η^/σ^Ε^-dependent P2*dnaK* regulated by HspR-associated inverted repeat (HAIR) in *C. glutamicum* (Pátek et al., 2013). Another possibility is that DNA in the promoter region is unwinded by RNAP holoenzyme carrying stress sigma factor and transcription bubble is formed. Then DNA may be exposed to bind RNAP carrying different sigma factor (e.g., σ^A^) to another close promoter.

The analysis of the *sigH*-*rshA* operon showed that the transcription of *sigH* is also rarely autoregulated (in only 7 *Corynebacterium* species). In contrast to *cseE*, upstream of the *rshA* gene, conserved stress promoters (GGAAT–N_18_–GTT) were recognized in 63 *Corynebacterium* species. We can infer from these sequence analyses that the transcription of *sigE* and *sigH* in response to stress conditions should therefore be regulated mostly by DNA-binding transcriptional factors. Moreover, a certain level of σ factors could be permanently present in the cell in an inactive form bound by the cognate anti-σ factor. The additional σ^Η^- or σ^Ε^-dependent promoter upstream of *rshA* or *cseE*, respectively, probably developed as a different strategy to ensure sufficient production of the anti-σ that can inhibit the σ factor when the stress is over (Busche et al., 2012).

Expression of the *sigE* gene is regulated by a two-component regulatory system in *M. tuberculosis* (Donà et al., 2008) and *S. coelicolor* (Hutchings et al., 2006). We detected inverted repeats in the upstream sequence of *sigE* in *C. glutamicum* (nt −41 to −31 relative to TSS), which might be a binding site for a transcriptional regulator. We tested the activity of the promoter fragment −42 to +5 and various shortened versions, including those in which the inverted repeats were missing, but did not find any significant effect of these deletions on the P1*sigE* activity (data not shown). Measurements in the *C. glutamicum* strain deficient in the two-component system CgtRS2 (homologous with the MprAB two-component system regulating *sigE* gene expression in *M. tuberculosis*; Bott and Brocker, 2012) also did not show any effect on of *sigE* promoter activity. We can thus conclude, that the expression of the *sigE* gene in *C. glutamicum* is most probably not controlled by two-component system.

Deletion of the anti-σ^Ε^gene *cseE* did not result in upregulation of the *sigE* gene, which is in agreement with the finding that *sigE* is transcribed from σ^Α^/σ^Β^-dependent promoters (Figure 4A). We previously observed an analogous situation with the *sigH* and *rshA* genes (Busche et al., 2012).

Interestingly, the transcription of *cseE* is driven from an σ^Η^/σ^Ε^-dependent promoter (Figure 4) which suggests the cross-regulation of σ^Η^ and σ^Ε^. No such phenomenon was observed in the transcription of the *C. glutamicum rshA* gene, which was transcribed from a σ^Η^-specific promoter. However, a weak transcription of *rshA* induced in the Δ*cseE* strain was detected by RNA-seq (Table 3). This might also reflect an effect of indirect σ^Η^/σ^Ε^ cross-regulation. We speculate that σ^Η^ and σ^Ε^ may compensate if one of the sigma factor is missing due to different growth or stress conditions or gene defect. In fact, all found σ^Ε^-dependent genes were partially transcribed with σ^Η^, at least in some growth phases, due to the overlapping promoter recognition specificity. This notion is supported by the fact that *sigH* or *sigE* deletion strains are viable.

An intricate network of tightly co-regulated σ^Β^, σ^Ε^, and σ^Η^ sigma factors is documented by the fact, that σ^Η^ and σ^Ε^ complementarily control the transcription of the *sigB* gene. It was possible to separately generate single *sig* deletions for each of these three genes; however, we were unable to isolate σ^Η^ + σ^Ε^ double mutant in the previous studies (not shown). This double deletion is probably lethal due to the fact that these two σ factors secure too many functions in stress responses and therefore cannot be replaced by any of the three remaining *C. glutamicum* ECF sigma factors (σ^C^, σ^D^, and σ^Μ^) which provide a limited number of activities. Moreover, σ^Η^ and σ^Ε^ drive the transcription of *sigB* that also controls a wide range of functions. The σ^Β^-σ^Ε^-σ^Η^ “sigma triangle” or “Big Three” thus forms the essential basis for maintaining *C. glutamicum* cell viability during transition and stationary growth phases and diverse types of stress conditions. The integration of σ^Β^, σ^Ε^ and σ^Η^ in an intricate regulatory network was also observed in *M. tuberculosis* (Raman et al., 2001; Dutta et al., 2010), *Salmonella* (Bang et al., 2005), and also most likely exists in *R. erythropolis* (Štěpánek et al., 2022).

## Data availability statement

The transcriptome sequencing raw data files are available in the ArrayExpress database (www.ebi.ac.uk/arrayexpress) under accession numbers E-MTAB-12457 and E-MTAB-12458.

## Author contributions

JK and MP conceived the project and led the studies performed in Bielefeld and Prague, respectively. TB, HD, LR, and VŠ carried out the most experiments. TB and HD carried out RNA-seq and processed the data. JH did the *in vitro* transcription assays. IB carried out *in silico* analyses. All authors analyzed the results. MP, TB, and JK drafted the initial manuscript. All authors contributed to the article and approved the submitted version.

## Funding

This work was supported by Grant 17-06991S from the Czech Science Foundation, Mobility Grant DAAD-18-11 from the Czech Academy of Sciences (CAS), and Deutscher Akademischer Austauschdienst.

## Conflict of interest

The authors declare that this work was carried out in the absence of any personal, professional, or financial relationships that could potentially be construed as a conflict of interest.

## Publisher’s note

All claims expressed in this article are solely those of the authors and do not necessarily represent those of their affiliated organizations, or those of the publisher, the editors and the reviewers. Any product that may be evaluated in this article, or claim that may be made by its manufacturer, is not guaranteed or endorsed by the publisher.
