# Supplementary material for: Overlapping SigH and SigE sigma factor regulons in Corynebacterium glutamicum
Source: Front Microbiol. 2023 Feb 28;13:1059649. doi: 10.3389/fmicb.2022.1059649 (PMC10012870; doi:10.3389/fmicb.2022.1059649)
Supplement: Supplementary file 1 [file Table_1.DOCX]

| Supplementary Table 1. Oligonucleotides used | | |
| --- | --- | --- |
| **Name** | **Sequence** | **Purpose** |
| PMSHCPEPRF | GGATTTTTTGGTTTGTGGAATAGGTGCACTGGCGGCTTGGTTGAAGTTTCAGGTGACG | Cloning of promoter P*mshC* from *C. glutamicum* ATCC 13032 into pEPR1 |
| PMSHCPEPRR | GATCCGTCACCTGAAACTTCAACCAAGCCGCCAGTGCACCTATTCCACAAACCAAAAAATCCTGCA | Cloning of promoter P*mshC* from *C. glutamicum* ATCC 13032 into pEPR1 |
| PMSHCP770F | AATTCGATTTTTTGGTTTGTGGAATAGGTGCACTGGCGGCTTGGTTGAAGTTTCAGGTGACA | Cloning of promoter P*mshC* from *C. glutamicum* ATCC 13032 into pRLG770 |
| PMSHCP770R | AGCTTGTCACCTGAAACTTCAACCAAGCCGCCAGTGCACCTATTCCACAAACCAAAAAATCG | Cloning of promoter P*mshC* from *C. glutamicum* ATCC 13032 into pRLG770 |
| PUVRD3PEPRF | GCTTTAACCGCTATCTGGAATGATTGATAGCTCCCAAGTGTTGTATCTATTCCAGTTG | Cloning of promoter P*uvrD3* from *C. glutamicum* ATCC 13032 into pEPR1 |
| PUVRD3PEPRR | GATCCAACTGGAATAGATACAACACTTGGGAGCTATCAATCATTCCAGATAGCGGTTAAAGCTGCA | Cloning of promoter P*uvrD3* from *C. glutamicum* ATCC 13032 into pEPR1 |
| PUVRD3P770F | AATTCCTTTAACCGCTATCTGGAATGATTGATAGCTCCCAAGTGTTGTATCTATTCCAGTTA | Cloning of promoter P*uvrD3* from *C. glutamicum* ATCC 13032 into pRLG770 |
| PUVRD3P770R | AGCTTAACTGGAATAGATACAACACTTGGGAGCTATCAATCATTCCAGATAGCGGTTAAAGG | Cloning of promoter P*uvrD3* from *C. glutamicum* ATCC 13032 into pRLG770 |
| SUFRF | ttctgcagtcgcatcattctacgc | Cloning of promoter P*sufR* from *C. glutamicum* ATCC 13032 into pEPR1 |
| SUFRR | gtgcaggatccaacaggatgagaaga | Cloning of promoter P*sufR* from *C. glutamicum* ATCC 13032 into pEPR1 |
| SUFRP770F | aattcgaagggattggacacgggaatggaattagggaacacttgtgttgtctaaaggtgaaaa | Cloning of promoter P*sufR* from *C. glutamicum* ATCC 13032 into pRLG770 |
| SUFRP770R | agctttttcacctttagacaacacaagtgttccctaattccattcccgtgtccaatcccttcg | Cloning of promoter P*sufR* from *C. glutamicum* ATCC 13032 into pRLG770 |
| PMCAPEPRF | GGGACGTTGGGTGTTTGGAATGTTCGAAGCGCCTGATGCGTTGGATGGAGAGTTGGAG | Cloning of promoter P*mca* from *C. glutamicum* ATCC 13032 into pEPR1 |
| PMCAPEPRR | GATCCTCCAACTCTCCATCCAACGCATCAGGCGCTTCGAACATTCCAAACACCCAACGTCCCTGCA | Cloning of promoter P*mca* from *C. glutamicum* ATCC 13032 into pEPR1 |
| PMCAP770F | AATTCGGACGTTGGGTGTTTGGAATGTTCGAAGCGCCTGATGCGTTGGATGGAGAGTTGGAA | Cloning of promoter P*mca* from *C. glutamicum* ATCC 13032 into pRLG770 |
| PMCAP770R | AGCTTTCCAACTCTCCATCCAACGCATCAGGCGCTTCGAACATTCCAAACACCCAACGTCCG | Cloning of promoter P*mca* from *C. glutamicum* ATCC 13032 into pRLG770 |
| cg0378 F | AACTGCAGAGCTGGGGTTTTCT | Cloning of promoter P*cg0378* from *C. glutamicum* ATCC 13032 into pEPR1 |
| cg0378 R | ACGGATCCCTTACTCCTACAAC | Cloning of promoter P*cg0378* from *C. glutamicum* ATCC 13032 into pEPR1 |
| P0378770F | AATTCgattccatttacgatggaacatttttgaagaatacctcgttgaatctagtgcaaA | Cloning of promoter P*cg0378* from *C. glutamicum* ATCC 13032 into pRLG770 |
| P0378770R | AGCTTTTGCACTAGATTCAACGAGGTATTCTTCAAAAATGTTCCATCGTAAATGGAATCG | Cloning of promoter P*cg0378* from *C. glutamicum* ATCC 13032 into pRLG770 |
| PAMTRF | TGAGGCTGCAGAGGTCTATTT | Cloning of promoter P*amtR* from *C. glutamicum* ATCC 13032 into pEPR1 |
| PAMTRR | CACGGGGATCCACGAGGC | Cloning of promoter P*amtR* from *C. glutamicum* ATCC 13032 into pEPR1 |
| AMTRP770F | AATTCTTGTCATCTAGGCCGCCTGCGGTGGAAACTTATGAGGGTCTTCCTTCGTTGGACATGGTGGTA | Cloning of promoter P*amtR* from *C. glutamicum* ATCC 13032 into pRLG770 |
| AMTRP770R | AGCTTTACCACCATGTCCAACGAAGGAAGACCCTCATAAGTTTCCACCGCAGGCGGCCTAGATGACAAG | Cloning of promoter P*amtR* from *C. glutamicum* ATCC 13032 into pRLG770 |
| CG1121PEPR1F | ATCCCTGCAGGCACCCAAAC | Cloning of promoter P*cg1121* from *C. glutamicum* ATCC 13032 into pEPR1 |
| CG1121PEPR1R | TCAATGGATCCTTCGTCGTCAATCAAGTTA | Cloning of promoter P*cg1121* from *C. glutamicum* ATCC 13032 into pEPR1 |
| CG1121P770F | AATTCAGTAAAGGTGTGAAAATAGTTCCTCACGTGGGGAACTATACTGATCCTTGATGCGTTAACTTGATTGACGA | Cloning of promoter P*cg1121* from *C. glutamicum* ATCC 13032 into pRLG770 |
| CG1121P770R | AGCTTCGTCAATCAAGTTAACGCATCAAGGATCAGTATAGTTCCCCACGTGAGGAACTATTTTCACACCTTTACTG | Cloning of promoter P*cg1121* from *C. glutamicum* ATCC 13032 into pRLG770 |
| PCG1277PEPRF | GGGAAATTCCACTCATGAACGCCTAGTCTACGGGAACCATTTCACCAGCGTGTACGTTGTAAATGTGAACG | Cloning of promoter P*cg1277* from *C. glutamicum* ATCC 13032 into pEPR1 |
| PCG1277PEPRR | GATCCGTTCACATTTACAACGTACACGCTGGTGAAATGGTTCCCGTAGACTAGGCGTTCATGAGTGGAATTTCCCTGCA | Cloning of promoter P*cg1277* from *C. glutamicum* ATCC 13032 into pEPR1 |
| Pcg1277p770F | AATTccactcatgaacgcctagtctacgggaaccatttcaccagcgtgtacgttgtaaatgtgaactga | Cloning of promoter P*cg1277* from *C. glutamicum* ATCC 13032 into pRLG770 |
| Pcg1277p770R | AGCTTCAGTTCACATTTACAACGTACACGCTGGTGAAATGGTTCCCGTAGACTAGGCGTTCATGAGTGG | Cloning of promoter P*cg1277* from *C. glutamicum* ATCC 13032 into pRLG770 |
| PCLGR770F | AATTCTTTCCAGGAAAACCGGGAACAAATTTTAGGGAAAGGGAGTTGAACCTAACGATA | Cloning of promoter P1*clgR* from *C. glutamicum* ATCC 13032 into pRLG770 |
| PCLGR770R | AGCTTATCGTTAGGTTCAACTCCCTTTCCCTAAAATTTGTTCCCGGTTTTCCTGGAAAG | Cloning of promoter P1*clgR* from *C. glutamicum* ATCC 13032 into pRLG770 |
| PCLGRP2MUTF | GTGACCATATTGAGTCGCAGTGACTCAAGTTTCCAGGAAAACCGGGAACAAATTTTAGGGAAAGGGAGTTGAACCTAACGAG | Cloning of promoter P1*clgR* from *C. glutamicum* ATCC 13032 into pEPR1 |
| PCLGRP2MUTR | GATCCTCGTTAGGTTCAACTCCCTTTCCCTAAAATTTGTTCCCGGTTTTCCTGGAAACTTGAGTCACTGCGACTCAATATGGTCACTGCA | Cloning of promoter P1*clgR* from *C. glutamicum* ATCC 13032 into pEPR1 |
| P3309F | GTCTGCAGTTTTGTGGGTTTTCTC | Cloning of promoter P*cg3309* from *C. glutamicum* ATCC 13032 into pEPR1 |
| P3309R | AGGGATCCTACAAAGGTGGTTGAA | Cloning of promoter P*cg3309* from *C. glutamicum* ATCC 13032 into pEPR1 |
| CG3309P770F | AATTCGCTTTTCGACGTCTCCCTCCTCCCGTGGGAACTTTCCTCTAACTAAGTGCGTTAAGGAAGGTAGAAA | Cloning of promoter P*cg3309* from *C. glutamicum* ATCC 13032 into pRLG770 |
| CG3309P770R | AGCTTTTCTACCTTCCTTAACGCACTTAGTTAGAGGAAAGTTCCCACGGGAGGAGGGAGACGTCGAAAAGCG | Cloning of promoter P*cg3309* from *C. glutamicum* ATCC 13032 into pRLG770 |
| PCG3309MUTHF | GTTTTGCTTTTCGACGTCTCCCTCCTCCCGTGGGAATAACTCTCTAACTAAGTGCGTTAAGGAAGGTAGAAG | Cloning of promoter P*cg3309*mut from *C. glutamicum* ATCC 13032 into pEPR1 |
| PCG3309MUTHR | GATCCTTCTACCTTCCTTAACGCACTTAGTTAGAGAGTTATTCCCACGGGAGGAGGGAGACGTCGAAAAGCAAAACTGCA | Cloning of promoter P*cg3309*mut from *C. glutamicum* ATCC 13032 into pEPR1 |
| cg3344 F | ATCTGCAGTGTTTCTTACTTTA | Cloning of promoter P*cg3344* from *C. glutamicum* ATCC 13032 into pEPR1 |
| cg3344 R | GAGGATCCGTGCGTCTTCGTCA | Cloning of promoter P*cg3344* from *C. glutamicum* ATCC 13032 into pEPR1 |
| P3344770F | AATTCcacttacttgatgcgggaacaaatttgaaggtttttcagttgctataggtatgacA | Cloning of promoter P*cg3344* from *C. glutamicum* ATCC 13032 into pRLG770 |
| P3344770R | AGCTTGTCATACCTATAGCAACTGAAAAACCTTCAAATTTGTTCCCGCATCAAGTAAGTGG | Cloning of promoter P*cg3344* from *C. glutamicum* ATCC 13032 into pRLG770 |
| TRXB1F | gtctgcagatcaatatccacaccct | Cloning of promoter P*trxB1* from *C. glutamicum* ATCC 13032 into pEPR1 |
| TRXB1R | gccggatcctgtgctctcaaatgt | Cloning of promoter P*trxB1* from *C. glutamicum* ATCC 13032 into pEPR1 |
| TRXB1P770F | aattcagaaaatccttggccgggaataactacagtccgctgaaagttggtctatatatagacca | Cloning of promoter P*trxB1* from *C. glutamicum* ATCC 13032 into pRLG770 |
| TRXB1P770R | agcttggtctatatatagaccaactttcagcggactgtagttattcccggccaaggattttctg | Cloning of promoter P*trxB1* from *C. glutamicum* ATCC 13032 into pRLG770 |
| PTRXB1MUTEF | GTGCCGGTCAATGAAGAAAATCCTTGGCCGGGAACTTTCACAGTCCGCTGAAAGTTGGTCTATATATAGACG | Cloning of promoter P*trxB1*mut from *C. glutamicum* ATCC 13032 into pEPR1 |
| PTRXB1MUTER | GATCCGTCTATATATAGACCAACTTTCAGCGGACTGTGAAAGTTCCCGGCCAAGGATTTTCTTCATTGACCGGCACTGCA | Cloning of promoter P*trxB1*mut from *C. glutamicum* ATCC 13032 into pEPR1 |
| PRSHAMUTEF | GCACTCGGAACTGTGATGTCCCGACTCCATCGTGGAACTTATCAGCTCCGAGGAATGTTAAAGGAAGTAGCGG | Cloning of promoter P*rshA*mut from *C. glutamicum* ATCC 13032 into pEPR1 |
| PRSHAMUTER | GATCCCGCTACTTCCTTTAACATTCCTCGGAGCTGATAAGTTCCACGATGGAGTCGGGACATCACAGTTCCGAGTGCTGCA | Cloning of promoter P*rshA*mut from *C. glutamicum* ATCC 13032 into pEPR1 |
| PCSEEPEPRF | GtcgtggtgcggacagggaacttatcacaggcgacatccgttttgagtagtaggtG | Cloning of promoter P2*cseE* from *C. glutamicum* ATCC 13032 into pEPR1 |
| PCSEEPEPRR | GATCCACCTACTACTCAAAACGGATGTCGCCTGTGATAAGTTCCCTGTCCGCACCACGACTGCA | Cloning of promoter P2*cseE* from *C. glutamicum* ATCC 13032 into pEPR1 |
| PCSEAMUTHF | GTGCATTATTTTGGGGTTCGTGGTGCGGACAGGGAAGAAAACACAGGCGACATCCGTTTTGAGTAGTAGGTG | Cloning of promoter P2*cseE*mut from *C. glutamicum* ATCC 13032 into pEPR1 |
| PCSEAMUTHR | GATCCACCTACTACTCAAAACGGATGTCGCCTGTGTTTTCTTCCCTGTCCGCACCACGAACCCCAAAATAATGCACTGCA | Cloning of promoter P2*cseE*mut from *C. glutamicum* ATCC 13032 into pEPR1 |
| PCSEA770F | AATTCtcgtggtgcggacagggaacttatcacaggcgacatccgttttgagtagtaggtA | Cloning of promoter P2*cseE* from *C. glutamicum* ATCC 13032 into pRLG770 |
| PCSEA770R | AGCTTACCTACTACTCAAAACGGATGTCGCCTGTGATAAGTTCCCTGTCCGCACCACGAG | Cloning of promoter P2*cseE* from *C. glutamicum* ATCC 13032 into pRLG770 |
| P1SIGEPEPRF | GTTCACAATTGCCAGAAGATGCACAGGATGTAATCTAGATTTCCCAAGTTCAGTGGGGCAAAATGACTTATATGAAG | Cloning of promoter P1*sigE* from *C. glutamicum* ATCC 13032 into pEPR1 |
| P1SIGEPEPRR | GATCCTTCATATAAGTCATTTTGCCCCACTGAACTTGGGAAATCTAGATTACATCCTGTGCATCTTCTGGCAATTGTGAACTGCA | Cloning of promoter P1*sigE* from *C. glutamicum* ATCC 13032 into pEPR1 |
| SIGETSP1P770F | aattcgcacaggatgtaatctagatttcccaagttcagtggggcaaaatgacttatatgaa | Cloning of promoter P1*sigE* from *C. glutamicum* ATCC 13032 into pRLG770 |
| SIGETSP1P770R | agctttcatataagtcattttgccccactgaacttgggaaatctagattacatcctgtgcg | Cloning of promoter P1*sigE* from *C. glutamicum* ATCC 13032 into pRLG770 |
| SIGEmutR-MF | GGGTACCGTGATGTCCCGTATTCACCG | Cloning of structural gene *sigE* from *C. glutamicum* ATCC 13032 into pEC-XT99A, mutation Arg → Met |
| SIGEmutR-MR | ATTTTCACTCCGAGGGTC | Cloning of structural gene *sigE* from *C. glutamicum* ATCC 13032 into pEC-XT99A, mutation Arg → Met |
| SIGHmutM-RF | CGGAACTGTGCGCTCCCGACTCCATC | Cloning of structural gene *sigH* from *C. glutamicum* ATCC 13032 into pEC-XT99A, mutation Met → Arg |
| SIGHmutM-RRa | AGTGGAACGTCCATGATCTCGG | Cloning of structural gene *sigH* from *C. glutamicum* ATCC 13032 into pEC-XT99A, mutation Met → Arg |
| PSIGEH5 | GACTGCAGTCGGCGGCGTCTTC | Cloning of promoter P*sigE* from *C. glutamicum* ATCC 13032 into pET2 |
| PSIGED5 | TCATCTCGGGATCCCTTTTTCATA | Cloning of promoter P*sigE* from *C. glutamicum* ATCC 13032 into pET2 |
| PRSEAHP | cactgcagttggtgtgttttctg | Cloning of promoter P*cseE* from *C. glutamicum* ATCC 13032 into pET2 |
| PRSEADB | ttggatcctcgcggtggtgtcgg | Cloning of promoter P*cseE* from *C. glutamicum* ATCC 13032 into pET2 |
