# Supplementary material for: Overlapping SigH and SigE sigma factor regulons in Corynebacterium glutamicum
Source: Front Microbiol. 2023 Feb 28;13:1059649. doi: 10.3389/fmicb.2022.1059649 (PMC10012870; doi:10.3389/fmicb.2022.1059649)
Supplement: Supplementary file 2 [file Table_2.DOCX]

Supplementary Table 2. List of identified SigE-upregulated genes as detected by RNA-seq with *C. glutamicum* RES167 and Δ*cseE* strains

| **Gene number** | **Gene name** | **Product** | **M-value UP (padj<=0.01 and m>=1)** |
| --- | --- | --- | --- |
| *cg0047* |  | Conserved hypothetical protein | 1.39 |
| *cg0075* |  | Conserved hypothetical protein | 1.37 |
| *cg0085* | *phoH1* | NYN ribonuclease and ATPase of PhoH family | 2.06 |
| *cg0092* |  | Putative membrane protein | 1.36 |
| *cg0103* | *crnT* | Putative drug efflux permease, MFS-type | 2.11 |
| *cg0104* | *codA* | cytosine deaminase | 2.36 |
| *cg0113* | *ureA* | Urease gamma subunit | 1.15 |
| *cg0114* | *ureB* | Urease beta subunit | 1.41 |
| *cg0129* | *putA* | Proline dehydrogenase/delta-1-pyrroline-5-carboxylate dehydrogenase | 1.12 |
| *cg0131* |  | Putative aldo-keto reductase | 1.31 |
| *cg0142* | *sixA* | Putative arabitol-1-phosphatase | 1.26 |
| *cg0152* |  | Hypothetical protein | 1.07 |
| *cg0154* |  | Putative hydrolase | 1.25 |
| *cg0155* |  | Conserved hypothetical protein, carbonic anhydrases/acetyltransferases (isoleucine patch superfamily) | 1.19 |
| *cg0166* |  | Conserved hypothetical protein, Ankyrin repeat | 1.11 |
| *cg0168* |  | Putative secondary chloramphenicol transporter, drug/metabolite transporter (DMT) superfamily | 2 |
| *cg0175* |  | Putative secreted protein | 1.11 |
| *cg0196* | *iolR* | Putative transcriptional regulator, GntR-family | 1.72 |
| *cg0197* | *iolC* | Putative 5-dehydro-2-deoxygluconokinase | 1.34 |
| *cg0201* | *iolB* | Uncharacterized enzyme involved in inositol metabolism | 1.09 |
| *cg0202* | *iolD* | Putative acetolactate synthase, large subunit | 1.26 |
| *cg0209* |  | Hypothetical protein | 1 |
| *cg0229* | *gltB* | Glutamate synthase (NADPH), large chain | 5.94 |
| *cg0230* | *gltD* | Glutamate synthase (NADPH), small chain | 4.66 |
| *cg0246* |  | Glycosyl transferase | 1.13 |
| *cg0253* |  | Putative 2Fe-2S ferredoxin | 1.23 |
| *cg0282* |  | CsbD family protein probably involved in stress response | 1.65 |
| *cg0297* |  | Conserved hypothetical protein, DUF149-family | 1.32 |
| *cg0315* | *brnE* | Secondary branched-chain amino acid efflux transporter, LIV-E family, small subunit | 1.09 |
| *cg0316* |  | Putative secreted protein | 2.03 |
| *cg0333* |  | Putative membrane protein | 1.09 |
| *cg0353* | *nth* | Putative endonuclease III | 1.13 |
| *cg0362* | *cg0362* | Conserved hypothetical protein | 1.78 |
| *cg0363* |  | Putative secretion ATPase | 2.81 |
| *cg0365* |  | Putative membrane protein | 2.2 |
| *cg0378* |  | Putative phage-associated protein | 1.98 |
| *cg0380* |  | Hypothetical protein | 1.89 |
| *cg0382* |  | Conserved hypothetical protein | 1.33 |
| *cg0387* |  | Putative NAD/mycothiol-dependent formaldehyde dehydrogenase | 1.75 |
| *cg0388* |  | Putative Zn-dependent hydrolase | 1.57 |
| *cg0396* |  | Putative glycosyl transferase | 1.08 |
| *cg0454* |  | Putative transcriptional regulator, TetR-family | 2.14 |
| *cg0455* |  | Putative multidrug efflux permease, MFS-type | 2.01 |
| *cg0456* |  | Putative multidrug efflux permease, MFS-type | 1.51 |
| *cg0505* |  | Putative ribosomal protein L7/L12 family | 1.06 |
| *cg0517* | *hemY* | Protoporphyrinogen oxidase | 1.06 |
| *cg0527* | *glyR* | Transcriptional activator of glyA, ArsR-family | 1 |
| *cg0528* |  | Putative secreted protein | 1.22 |
| *cg0530* |  | Hypothetical protein | 1.03 |
| *cg0532* |  | Putative glycosyltransferase | 1.31 |
| *cg0562* | *nusG* | Transcription antitermination protein NusG | 1.51 |
| *cg0588* |  | Hypothetical protein | 1.42 |
| *cg0612* | *dkg* | Putative aldo/keto reductase, related to diketogulonate reductase | 1.45 |
| *cg0662* |  | Conserved hypothetical protein, putative FAD/FMN-containing dehydrogenase | 1.13 |
| *cg0690* | *groES* | 10kDa chaperonin | 1.79 |
| *cg0691* | *groEL'* | 60kDa chaperonin, putative pseudogene (N-terminal fragment) | 1.92 |
| *cg0692* | *tnp1c(ISCg1c)* | Transposase | 2.24 |
| *cg0709* | *cgtR7* | Two-component system, transcriptional response regulator | 1.03 |
| *cg0742* |  | Putative integral membrane protein | 1.33 |
| *cg0763* | *mdh2* | Putative malate/L-lactate dehydrogenase | 1.73 |
| *cg0780* |  | Membrane protein, ribonuclease BN-like family | 1.03 |
| *cg0788* | *pmmB* | Phosphoglucomutase/phosphomannomutase | 1.27 |
| *cg0795* |  | Putative oxidoreductase | 1.21 |
| *cg0796* | *prpD1* | Putative (2-methyl) citrate dehydratase | 1.17 |
| *cg0797* | *prpB1* | Putative (methyl)isocitrate lyase | 1.03 |
| *cg0815* |  | Putative membrane protein | 1.21 |
| *cg0829* |  | Conserved hypothetical protein, glyoxylase-family | 1.18 |
| *cg0859* |  | Conserved hypothetical protein | 1.31 |
| *cg0866* |  | Conserved hypothetical protein | 1.65 |
| *cg0867* |  | Putative ribosome-associated protein Y (PSrp-1) | 1.46 |
| *cg0876* | *sigH* | RNA polymerase sigma factor, ECF-family | 1.38 |
| *cg0877* | *rshA* | anti-sigma factor | 1.47 |
| *cg0882* |  | Conserved hypothetical protein | 1.18 |
| *cg0911* |  | Putative inositol monophosphatase | 1.2 |
| *cg0960* |  | Hypothetical protein | 1.79 |
| *cg0963* |  | Hypothetical protein | 1.78 |
| *cg0976* | *pcrA* | Putative ATP-dependent DNA helicase | 1 |
| *cg1028* |  | Putative restriction-modification system methylase | 1.11 |
| *cg1033* |  | Putative secondary Cd2+ transporter, cadmium resistance (CadD) family | 1.19 |
| *cg1045* |  | Conserved hypothetical protein | 1.17 |
| *cg1061* | *urtA* | ABC-type putative branched-chain amino acid transporter, substrate-binding lipoprotein | 2.35 |
| *cg1062* | *urtB* | ABC-type putative branched-chain amino acid transporter, permease subunit | 1.58 |
| *cg1064* | *urtC* | ABC-type putative branched-chain amino acid transporter, permease subunit | 1.42 |
| *cg1066* | *urtE* | ABC-type putative branched-chain amino acid transporter, ATPase subunit | 1.66 |
| *cg1068* |  | Putative oxidoreductase | 1.29 |
| *cg1080* |  | Putative multicopper oxidase | 1.11 |
| *cg1106* |  | conserved hypothetical protein | 1.82 |
| *cg1107* |  | Putative pyrophosphatase | 1.63 |
| *cg1119* |  | Conserved hypothetical protein | 2.5 |
| *cg1121* |  | Permease, MFS-type | 2.58 |
| *cg1139* |  | Allophanate hydrolase subunit 2 | 1.26 |
| *cg1140* |  | Allophanate hydrolase subunit 1 | 1 |
| *cg1141* |  | Conserved hypothetical protein, UPF0271-family | 1.35 |
| *cg1142* |  | Putative Mn2+ transporter, metal ion (Mn2+-iron) transporter (Nramp) family | 1.69 |
| *cg1191* |  | Hypothetical protein | 1.16 |
| *cg1195* |  | Putative permease, sulfate permease (SulP) family | 1.13 |
| *cg1204* |  | Conserved hypothetical protein, similar to 2,3-PDG dependent phosphoglycerate mutase | 1.15 |
| *cg1206* |  | Conserved hypothetical protein | 1.6 |
| *cg1207* |  | ABC-type transporter, ATPase subunit | 1.02 |
| *cg1208* |  | Conserved hypothetical protein | 1.43 |
| *cg1213* | *tnp1a(ISCg1a)* | Transposase | 2.32 |
| *cg1222* | *lplA* | Lipoate-protein ligase A | 1.7 |
| *cg1232* |  | Conserved hypothetical protein, LmbE-family | 1.1 |
| *cg1261* |  | Lysine decarboxylase family protein | 1.74 |
| *cg1262* | *folP2* | Dihydropteroate synthase | 1.3 |
| *cg1263* |  | Glycosyltransferase, involved in cell wall biogenesis | 1.33 |
| *cg1273* |  | Putative twin arginine targeting (Tat) Preprotein translocase subunit | 3.1 |
| *cg1277* |  | Conserved putative membrane protein | 2.68 |
| *cg1278* |  | Conserved putative secreted protein | 1.88 |
| *cg1284* | *lipT* | Putative carboxylesterase, type B | 1.2 |
| *cg1296* |  | Conserved hypothetical protein, putative non-ribosomal peptide synthetase module | 2.43 |
| *cg1310* | *tfdF* | Putative maleylacetate reductase | 1.5 |
| *cg1311* |  | Hydroxyquinol 1,2-dioxygenase | 1.54 |
| *cg1327* |  | Putative transcriptional regulator, Crp-family | 1.53 |
| *cg1328* |  | Putative heavy-metal ion transporting P-type ATPase | 1.1 |
| *cg1329* | *ctpC* | Putative Cd2+ transporting P-type ATPase | 1.06 |
| *cg1337* | *hom* | Homoserine dehydrogenase | 1.26 |
| *cg1338* | *thrB* | Homoserine kinase | 1.68 |
| *cg1370* |  | Conserved hypothetical protein | 1.37 |
| *cg1371* |  | Putative nuclease, RecB-family | 1.03 |
| *cg1386* | *fixA* | Putative electron transfer flavoprotein, beta subunit | 1.26 |
| *cg1402* |  | Conserved hypothetical protein | 1.08 |
| *cg1410* | *rbsR* | Transcriptional repressor of the ribose importer RbsACBD, LacI-family | 1.15 |
| *cg1423* |  | Putative oxidoreductase, aldo/keto reductase family | 1.12 |
| *cg1447* |  | Putative secondary Co2+/Zn2+/Cd2+ efflux transporter, cation diffusion facilitator (CDF) family | 1.14 |
| *cg1452* |  | Hypothetical protein | 1.06 |
| *cg1474* |  | Hypothetical protein | 1.35 |
| *cg1524* |  | Putative membrane protein | 1.12 |
| *cg1545* | *uriT* | Putative multidrug efflux permease, MFS-type | 1.21 |
| *cg1546* | *rbsK1* | Putative ribokinase | 1.33 |
| *cg1547* | *uriR* | Transcriptional regulator of the uriR operon | 1.05 |
| *cg1548* |  | Conserved hypothetical protein | 1.52 |
| *cg1549* |  | Hypothetical protein | 1.39 |
| *cg1553* | *qor2* | quinone oxidoreductase involve in disulfide stress response | 1.02 |
| *cg1606* | *pyrG* | CTP synthetase | 1.06 |
| *cg1607* |  | Putative NTP pyrophosphohydrolase | 1.07 |
| *cg1610* | *parA2* | Putative chromosome partitioning ATPase | 1.11 |
| *cg1645* |  | SAM-dependent methyltransferase | 1.15 |
| *cg1648* |  | Putative transcriptional regulator, RpiR-family | 1.02 |
| *cg1652* | *pctA* | ABC-type alkylphosphonate transporter, substrate-binding lipoprotein (TC 3.A.1.9.1) | 2.06 |
| *cg1653* | *pgp1* | Putative phosphoglycolate phosphatase | 1.93 |
| *cg1657* | *ufaA* | Putative cyclopropane-fatty-acyl-phospholipid synthase | 1.55 |
| *cg1675* |  | Putative membrane protein | 1.01 |
| *cg1733* |  | Conserved hypothetical protein | 1.23 |
| *cg1740* |  | Putative nucleoside-diphosphate-sugar epimerase | 1.86 |
| *cg1758* |  | Putative membrane protein | 1.36 |
| *cg1770* |  | DNA/RNA helicase, superfamily II-typ | 1.16 |
| *cg1783* | *soxA'* | Putative oxidase, pseudogene (N-terminal fragment) | 2.41 |
| *cg1784* | *ocd* | Putative ornithine cyclodeaminase | 3.61 |
| *cg1785* | *amt* | Putative secondary ammonium transporter, Amt-family | 3.25 |
| *cg1792* |  | Putative transcriptional regulator, WhiB-family | 1.03 |
| *cg1794* |  | Conserved hypothetical protein, P-loop ATPase protein family | 1.01 |
| *cg1802* | *fmu* | Ribosomal RNA small subunit methyltransferase B | 1.16 |
| *cg1803* | *fmt* | Methionyl-tRNA formyltransferase | 1.35 |
| *cg1804* | *def2* | Polypeptide deformylase | 1.64 |
| *cg1839* |  | Conserved hypothetical protein, ATPase related to the helicase subunit of the Holliday junction resolvase | 1.07 |
| *cg1869* | *ruvB* | Holliday junction resolvasome helicase subunit | 1.05 |
| *cg1870* | *ruvA* | Holliday junction resolvasome DNA-binding subunit | 1.68 |
| *cg1871* | *ruvC* | Crossover junction endodeoxyribonuclease | 1.58 |
| *cg1874* |  | Putative membrane protein | 2.46 |
| *cg1890* |  | Hypothetical protein | 1.16 |
| *cg1895* |  | Putative secreted protein | 1.53 |
| *cg1896* |  | Putative secreted protein | 1.24 |
| *cg1918* |  | Putative secreted protein | 1.18 |
| *cg1925* |  | Hypothetical protein | 1.45 |
| *cg1934* |  | Hypothetical protein | 1.77 |
| *cg1944* |  | Hypothetical protein | 1.76 |
| *cg1949* |  | Hypothetical protein | 1.12 |
| *cg1966* |  | Hypothetical protein | 1.85 |
| *cg2042* |  | Putative secreted protein | 1.65 |
| *cg2088* |  | Hypothetical protein | 1.34 |
| *cg2094* |  | Hypothetical protein | 2.65 |
| *cg2102* | *sigB* | RNA polymerase sigma factor rpoD (Sigma-A) | 1.38 |
| *cg2110* |  | Putative membrane protein | 1.15 |
| *cg2112* | *nrdR* | transcriptional regulator of deoxyribonucleotide synthesis, YbaD-family | 1.11 |
| *cg2115* | *sugR* | Transcriptional regulator, DeoR-family | 1.28 |
| *cg2116* |  | Putative phosphofructokinase | 1.1 |
| *cg2131* |  | Hypothetical protein | 1.31 |
| *cg2152* | *clgR* | Transcriptional activator of Clp protease genes | 1.94 |
| *cg2153* |  | Conserved hypothetical protein, CinA-like protein | 1.74 |
| *cg2154* | *pgsA2* | CDP-diacylglycerol--glycerol-3-phosphate 3-phosphatidyltransferase | 1.43 |
| *cg2161* | *dapA* | Dihydrodipicolinate synthase | 1.09 |
| *cg2171* | *pptA* | Putative phosphopantheteinyl transferase, iron-chelating complex subunit | 1.14 |
| *cg2201* |  | Two-component system, sensory histidine kinase | 1.04 |
| *cg2213* |  | ABC-type putative multidrug transporter, ATPase subunit | 1.02 |
| *cg2229* |  | Putative excinuclease ATPase subunit | 1.41 |
| *cg2243* |  | Putative secondary Na+:2-oxoglutarate/malate/di-,tricarboxylate symporter, divalent anion:Na+ symporter (DASS) family | 1.17 |
| *cg2260* | *glnK* | Nitrogen regulatory protein PII | 1.54 |
| *cg2261* | *amtB* | Putative secondary ammonium transporter, Amt-family | 2.95 |
| *cg2265* | *smc* | Chromosome segregation ATPase | 1.21 |
| *cg2266* |  | Putative acylphosphatase | 1.62 |
| *cg2267* |  | Putative membrane protein | 1.95 |
| *cg2279* |  | ABC-type multidrug/protein/lipid transporter, permease subunit and ATPase subunit | 1.27 |
| *cg2309* |  | Putative transcriptional regulator, TetR-family | 1.28 |
| *cg2344* |  | Cystathionine beta-synthase-like protein | 1.18 |
| *cg2370* | *ftsW* | Bacterial cell division membrane protein | 1.01 |
| *cg2376* |  | Putative secreted protein | 1.32 |
| *cg2377* | *mraW* | S-adenosylmethionine-dependent methyltransferase involved in cell envelope biogenesis | 1.3 |
| *cg2378* | *mraZ* | Putative MraZ protein | 1.77 |
| *cg2380* |  | Putative membrane protein | 2.59 |
| *cg2382* |  | Putative N-acetyltransferase, GCN5-related | 1.45 |
| *cg2392* |  | Conserved hypothetical protein | 1 |
| *cg2395* |  | Putative secreted or membrane protein | 1.5 |
| *cg2400* |  | Putative glycosyltransferase | 1.16 |
| *cg2431* |  | Conserved hypothetical protein, contains double-stranded beta-helix domain | 1.3 |
| *cg2432* |  | Conserved hypothetical protein, MUTT/NUDIX-family | 1.13 |
| *cg2442* |  | Putative permease, MFS-type, central region - putative pseudogene | 1.33 |
| *cg2443* |  | Permease of the major facilitator superfamily, C-terminal region - putative pseudogene | 2.1 |
| *cg2444* |  | Hypothetical protein | 1.58 |
| *cg2451* |  | Conserved hypothetical protein | 1.74 |
| *cg2457* |  | Conserved hypothetical protein | 1.09 |
| *cg2478* |  | Putative penicillin binding protein | 1.19 |
| *cg2489* | *dnaG* | DNA primase | 1.36 |
| *cg2516* | *hrcA* | Putative transcriptional regulator, HrcA-family | 1.12 |
| *cg2542* |  | Putative secondary malonate transporter, auxin efflux carrier (AEC) family | 1.26 |
| *cg2543* | *glcD* | Putative (S)-2-hydroxy-acid oxidase | 1.47 |
| *cg2554* | *rbsK2* | Ribokinase | 1.02 |
| *cg2587* |  | Phosphoglycerate dehydrogenase or related dehydrogenase | 1.67 |
| *cg2591* | *dkgA* | Putative 2,5-diketo-D-gluconic acid reductase | 2.42 |
| *cg2600* | *tnp1d(ISCg1d)* | Transposase | 2.37 |
| *cg2604* |  | Putative secreted or membrane protein | 1.47 |
| *cg2605* |  | Putative acetyltransferase | 1.68 |
| *cg2615* |  | Putative transcriptional regulator, PadR-family | 1.51 |
| *cg2619* |  | Putative secondary malonate transporter, auxin efflux carrier (AEC) family | 1.06 |
| *cg2657* |  | Putative membrane protein, putative pseudogen | 2.25 |
| *cg2679* |  | putative dehydrogenase | 1.32 |
| *cg2685* |  | Putative short-chain dehydrogenase/reductase | 1.34 |
| *cg2689* | *glbO* | Putative Hemoglobin-like protein | 1.29 |
| *cg2715* |  | Conserved hypothetical protein | 1.48 |
| *cg2725* | *tnp1b(ISCg1b)* | Transposase | 2.25 |
| *cg2734* | *pncA* | Putative nicotinamidase/pyrazinamidase | 1.42 |
| *cg2738* | *ppt1* | Holo-[acyl-carrier-protein] synthase | 1.12 |
| *cg2758* |  | Conserved putative membrane protein | 1.4 |
| *cg2805* | *psp4* | Putative secreted protein | 1.29 |
| *cg2809* |  | Putative membrane protein | 1.09 |
| *cg2835* |  | Putative acetyltransferase | 1.9 |
| *cg2847* | *mshD* | Putative 1-D-myo-inosityl-2-(L-cysteinyl)amido-2-deoxy-alpha-D- glucopyranoside N-acetyltransferase | 1.93 |
| *cg2861* |  | Putative membrane protein, hemolysin III homolog | 1.15 |
| *cg2867* | *gpx* | Putative mycothiol peroxidase, GSH peroxidase family | 1.07 |
| *cg2875* |  | Hypothetical protein | 1.07 |
| *cg2891* | *pqo* | Pyruvate:quinone oxidoreductase | 1.53 |
| *cg2893* |  | Putative multidrug efflux permease, MFS-type | 2.45 |
| *cg2894* |  | Putative transcriptional regulator, TetR family | 1.96 |
| *cg2953* |  | Putative 4-hydroxybenzaldehyde dehydrogenase | 1.08 |
| *cg2958* | *butA* | L-2,3-Butanediol dehydrogenase/acetoin reductase | 2.09 |
| *cg2962* |  | Hypothetical protein, uncharacterized enzyme involved in biosynthesis of extracellular polysaccharides | 3.1 |
| *cg2968* |  | Conserved hypothetical protein | 1.36 |
| *cg2973* |  | Conserved hypothetical protein | 3.15 |
| *cg2978* |  | Putative membrane protein | 1.27 |
| *cg2979* | *folK* | 2-Amino-4-hydroxy-6- hydroxymethyldihydropteridine diphosphokinase | 1.1 |
| *cg2981* | *folX* | Dihydroneopterin aldolase | 1.08 |
| *cg2982* | *folP1* | Dihydropteroate synthase | 1.12 |
| *cg2999* |  | Putative ferredoxin reductase | 1.17 |
| *cg3000* |  | Putative thiosulfate sulfurtransferase | 2.32 |
| *cg3004* | *gabD1* | Succinate-semialdehyde dehydrogenase (NAD(P)(+)) | 1.84 |
| *cg3011* | *groEL* | Chaperonin Cpn60 (60Kd subunit) | 1.52 |
| *cg3039* |  | Putative Na+/phosphate symporter, phosphate:Na+ symporter (PNaS) family | 1.85 |
| *cg3073* | *sseA1* | Thiosulfate sulfurtransferase | 1.45 |
| *cg3078* |  | Hypothetical protein | 2.1 |
| *cg3079* | *clpB* | Putative ATP-dependent protease (heat shock protein) | 2.13 |
| *cg3084* |  | Putative flavoprotein involved in K+ transport | 1.13 |
| *cg3090* |  | Conserved hypothetical protein | 1.06 |
| *cg3092* |  | Putative 2-polyprenylphenol hydroxylase or related flavodoxin oxidoreductase | 1.22 |
| *cg3095* |  | Conserved hypothetical protein | 1.04 |
| *cg3097* | *hspR* | Putative transcriptional regulator, MerR-family | 1.8 |
| *cg3098* | *dnaJ* | Chaperone DnaJ, heat shock protein | 1.78 |
| *cg3099* | *grpE* | Chaperone GrpE, heat shock protein | 2.47 |
| *cg3100* | *dnaK* | Chaperone DnaK, heat shock protein | 2.18 |
| *cg3119* | *fpr2* | Ferredoxin--NADP(+) reductase | 2.41 |
| *cg3120* |  | Conserved hypothetical protein | 1.15 |
| *cg3131* |  | Acetylornithine deacetylase or related deacylase | 1.38 |
| *cg3169* | *pck* | Phosphoenolpyruvate carboxykinase (GTP) | 1.17 |
| *cg3204* |  | Conserved hypothetical protein | 1.17 |
| *cg3215* | *glpQ1* | Glycerophosphodiester phosphodiesterase | 1.12 |
| *cg3253* | *mcbR* | Global transcriptional repressor of sulfur metabolism, TetR-family | 1.07 |
| *cg3254* |  | Putative membrane protein | 1.17 |
| *cg3271* |  | SAM-dependent methyltransferase | 1.01 |
| *cg3272* |  | Putative membrane protein | 1.77 |
| *cg3273* |  | Hypothetical protein | 1.95 |
| *cg3278* | *tnp20a(ISCg20a)* | Transposase, putative pseudogene | 1.52 |
| *cg3284* | *cgtS9* | Two-component system, sensory histidine kinase | 1.13 |
| *cg3285* | *cgtR9* | Two-component system, transcriptional response regulator | 2.19 |
| *cg3290* |  | Putative oxidoreductase | 1.93 |
| *cg3300* |  | Putative Cu2+ transporting P-type ATPase | 1.65 |
| *cg3309* |  | Putative secreted protein | 2.27 |
| *cg3332* | *qor* | NADPH:quinone reductase | 1.21 |
| *cg3339* | *merA* | Putative Hg2+ reductase | 1.25 |
| *cg3348* |  | Putative plasmid maintenance system antidote protein | 1.33 |
| *cg3357* | *trpP* | Permease, tryptophan-specific | 1.48 |
| *cg3370* |  | Putative NADH-dependent flavin oxidoreductase | 1.46 |
| *cg3373* | *cyeR* | Redox-sensing transcriptional repressor, ArsR-family | 1.66 |
| *cg3375* |  | Predicted nucleoside-diphosphate-sugar epimerase | 1.28 |
| *cg3396* |  | Putative membrane protein, stomatin/prohibitin homolog-like | 1.17 |
| *cg3397* |  | Hypothetical protein | 1.57 |
| *cg4001* | *ssrA* | SsrA peptide of tmRNA | 1.33 |
| *cg4010* |  | putative LacI-type transcriptional regulator, putative pseudogene (5'-end) | 1.03 |
