# Supplementary material for: Overlapping SigH and SigE sigma factor regulons in Corynebacterium glutamicum
Source: Front Microbiol. 2023 Feb 28;13:1059649. doi: 10.3389/fmicb.2022.1059649 (PMC10012870; doi:10.3389/fmicb.2022.1059649)
Supplement: Supplementary file 3 [file Table_3.DOCX]

Supplementary Table 3. List of identified SigE-downregulated genes as detected by RNA-seq with *C. glutamicum* RES167 and Δ*cseE* strains

| **Gene number** | **Gene name** | **Product** | **M-value DOWN (padj<=0.01 and m<=-1)** |
| --- | --- | --- | --- |
| *cg0015* | *gyrA* | DNA gyrase subunit A | -1.41 |
| *cg0016* |  | Putative integral membrane protein | -1.42 |
| *cg0044* |  | ABC-type putative sugar transporter, substrate-binding lipoprotein | -1.07 |
| *cg0046* |  | ABC-type putative sugar transporter, ATPase subunit | -1.66 |
| *cg0088* | *citP* | Putative secondary Mg2+/H+:citrate transporter, CitMHS-family | -2.72 |
| *cg0095* | *bioB* | Biotin synthase | -1.12 |
| *cg0096* |  | Conserved hypothetical protein | -1.36 |
| *cg0097* |  | Conserved hypothetical protein, putative zinc finger | -1.36 |
| *cg0107* |  | Putative secreted protein | -1.92 |
| *cg0148* | *panC* | Pantoate--beta-alanine ligase | -1.52 |
| *cg0161* |  | Putative membrane protein | -1.49 |
| *cg0165* |  | Putative membrane protein | -1.31 |
| *cg0173* |  | Conserved hypothetical protein | -1.77 |
| *cg0178* | *hrpB* | ATP-dependent RNA helicase | -1.05 |
| *cg0179* |  | Putative membrane protein | -2.65 |
| *cg0228* |  | Two-component system, sensory histidine kinase, putative pseudogene | -1.05 |
| *cg0231* |  | Putative membrane protein | -3.65 |
| *cg0232* |  | Putative secreted protein | -1.18 |
| *cg0235* | *emb* | arabinofuranosyltransferase | -1.71 |
| *cg0249* |  | ABC-type putative polysaccharide transporter, permease subunit | -1.37 |
| *cg0279* | *tyrA* | Putative prephenate dehydrogenase | -1.73 |
| *cg0286* |  | Conserved putative membrane protein | -1.79 |
| *cg0293* |  | Hypothetical protein | -1.46 |
| *cg0304* |  | Putative membrane protein | -1.46 |
| *cg0323* | *mnhE* | Putative Na+/H+ antiporter, MnhE subunit | -1.31 |
| *cg0334* |  | Putative secreted protein, probable acting as esterase | -1.55 |
| *cg0336* | *ponA* | Putative penicillin-binding protein 1B | -1.2 |
| *cg0341* | *fadD1* | Putative acyl-CoA synthase | -1.9 |
| *cg0350* | *glxR* | Transcriptional regulator, Crp-family | -1.15 |
| *cg0404* |  | Conserved hypothetical protein, nitroreductase-family | -1.38 |
| *cg0405* |  | ABC-type putative iron(III) dicitrate transporter, substrate-binding lipoprotein | -3.26 |
| *cg0444* | *ramB* | Transcriptional regulator, MerR-family | -1.32 |
| *cg0446* | *sdhA* | Succinate dehydrogenase, subunit A | -1.73 |
| *cg0447* | *sdhB* | Succinate dehydrogenase, subunit B | -1.46 |
| *cg0466* |  | Conserved putative secreted protein | -1.42 |
| *cg0477* |  | Hypothetical protein | -1.39 |
| *cg0483* | *cgtS4* | Two-component system, sensory histidine kinase | -1.1 |
| *cg0493* |  | Hypothetical protein | -1.58 |
| *cg0494* |  | Conserved hypothetical protein | -1.58 |
| *cg0504* | *aroE* | Putative shikimate 5-dehydrogenase | -1.3 |
| *cg0534* |  | Putative integral membrane protein | -3.44 |
| *cg0535* |  | Putative ketoglutarate semialdehyde dehydrogenase | -1.38 |
| *cg0536* |  | Putative 5-dehydro-4-deoxyglucarate dehydratase | -1.31 |
| *cg0544* |  | Putative membrane protein | -5.07 |
| *cg0545* | *pitA* | Putative phosphate/sulfate transporter, inorganic phosphate transporter (PiT) family | -2.64 |
| *cg0563* | *rplK* | 50S ribosomal protein L11 | -3.83 |
| *cg0564* | *rplA* | 50S ribosomal protein L1 | -3.76 |
| *cg0572* | *rplJ* | 50S ribosomal protein L10 | -2.85 |
| *cg0573* | *rplL* | Putative 50S ribosomal protein L7/L12 | -2.4 |
| *cg0576* | *rpoB* | DNA-directed RNA polymerase, beta chain | -2.21 |
| *cg0577* | *rpoC* | DNA-directed RNA polymerase, beta' chain | -1.58 |
| *cg0581* | *rpsL* | 30S ribosomal protein S12 | -1.87 |
| *cg0582* | *rpsG* | 30S ribosomal protein S7 | -2.1 |
| *cg0583* | *fusA* | Elongation factor G | -1.1 |
| *cg0589* |  | ABC-type putative iron-siderophore transporter, ATPase subunit | -2.85 |
| *cg0593* | *rpsJ* | 30S ribosomal protein S10 | -1.86 |
| *cg0594* | *rplC* | 50S ribosomal protein L3 | -2.35 |
| *cg0596* | *rplD* | 50S ribosomal protein L4 | -2.78 |
| *cg0597* | *rplW* | 50S ribosomal protein L23 | -3.41 |
| *cg0598* | *rplB* | 50S ribosomal protein L2 | -3.1 |
| *cg0599* | *rpsS* | 30S ribosomal protein S19 | -3.09 |
| *cg0600* | *rplV* | 50S ribosomal protein L22 | -2.96 |
| *cg0601* | *rpsC* | 30S ribosomal protein S3 | -2.39 |
| *cg0602* | *rplP* | 50S ribosomal protein L16 | -2.5 |
| *cg0603* | *rpmC* | 50S ribosomal protein L29 | -2.62 |
| *cg0604* | *rpsQ* | 30S ribosomal protein S17 | -1.79 |
| *cg0607* |  | Putative secreted protein | -1.46 |
| *cg0608* | *rplN* | 50S ribosomal protein L14 | -3.73 |
| *cg0609* | *rplX* | 50S ribosomal protein L24 | -3.52 |
| *cg0610* | *rplE* | 50S ribosomal protein L5 | -3.57 |
| *cg0616* | *fdhD* | Putative formate dehydrogenase, FdhD-family | -1.45 |
| *cg0617* |  | Hypothetical protein | -1.44 |
| *cg0621* |  | Putative integral membrane protein | -2.5 |
| *cg0622* |  | ABC-type putative cobalt transporter, ATPase subunit | -2.17 |
| *cg0623* |  | ABC-type putative cobalt transporter, permease subunit | -2.23 |
| *cg0624* |  | Putative secreted oxidoreductase | -1.42 |
| *cg0625* |  | Putative secreted protein | -1.53 |
| *cg0628* | *rpsH* | 30S ribosomal protein S8 | -2.55 |
| *cg0629* | *rplF* | 50S ribosomal protein L6 | -3.08 |
| *cg0630* | *rplR* | 50S ribosomal protein L18 | -3.23 |
| *cg0631* | *rpsE* | 30S ribosomal protein S5 | -2.94 |
| *cg0632* | *rpmD* | 50S ribosomal protein L30 | -3.09 |
| *cg0634* | *rplO* | 50S ribosomal protein L15 | -2.55 |
| *cg0637* | *betB* | Putative betaine aldehyde dehydrogenase (BADH) | -1.93 |
| *cg0639* |  | Putative ferredoxin reductase | -1.67 |
| *cg0645* | *cytP* | Putative cytochrome P450 | -2.81 |
| *cg0651* | *infA* | Translation initiation factor IF-1 | -2.51 |
| *cg0652* | *rpsM* | 30S ribosomal protein S13 | -4.11 |
| *cg0653* | *rpsK* | 30S ribosomal protein S11 | -3.66 |
| *cg0654* | *rpsD* | 30S ribosomal protein S4 | -3.82 |
| *cg0655* | *rpoA* | DNA-directed RNA polymerase, alpha subunit | -3.99 |
| *cg0656* | *rplQ* | 50S ribosomal protein L17 | -3.79 |
| *cg0664* |  | Putative membrane protein | -1.01 |
| *cg0673* | *rplM* | 50S ribosomal protein L13 | -2.36 |
| *cg0674* | *rpsI* | 30S ribosomal protein S9 | -2.29 |
| *cg0718* | *crtYf* | C50 carotenoid epsilon cyclase | -1.62 |
| *cg0719* | *crtYe* | C50 carotenoid epsilon cyclase | -1.98 |
| *cg0720* | *crtI2* | Phytoene dehydrogenase (desaturase) | -1.83 |
| *cg0721* | *crtB2* | Phytoene synthetase | -1.34 |
| *cg0736* | *metN* | ABC-type methionine transporter, ATPase subunit (TC 3.A.1.24.1) | -1.32 |
| *cg0748* |  | ABC-type putative iron-siderophore transporter, substrate-binding lipoprotein | -1.5 |
| *cg0749* | *spoU* | Putative tRNA/rRNA methyltransferase | -2.6 |
| *cg0755* | *metY* | O-Acetylhomoserine sulfhydrylase | -1.07 |
| *cg0759* | *prpD2* | 2-Methylcitrate dehydratase, involved in propionate catabolism | -1.73 |
| *cg0760* | *prpB2* | Methylisocitrate lyase, involved in propionate catabolism | -1.68 |
| *cg0762* | *prpC2* | 2-Methylcitrate synthase, involved in propionate catabolism | -2.08 |
| *cg0774* |  | Putative membrane protein | -1.3 |
| *cg0783* |  | Conserved hypothetical protein | -2.58 |
| *cg0786* | *upp* | Putative uracil phosphoribosyltransferase | -1.04 |
| *cg0791* | *pyc* | Pyruvate carboxylase | -1.18 |
| *cg0802* | *accBC* | Biotin carboxylase/biotin carboxyl carrier protein | -1.63 |
| *cg0808* | *wbpC* | Conserved putative membrane protein | -1.08 |
| *cg0811* | *dtsR2* | Acetyl/propionyl-CoA carboxylase, beta chain | -1.41 |
| *cg0816* | *purK* | Phosphoribosylaminoimidazole carboxylase, ATPase subunit | -1.48 |
| *cg0817* | *kup* | Putative secondary K+ uptake permease, K+ uptake permease (KUP) family | -1.34 |
| *cg0831* | *tusG* | ABC-type trehalose transporter, permease subunit | -1.06 |
| *cg0832* | *tusF* | ABC-type trehalose transporter, permease subunit | -1.45 |
| *cg0834* | *tusE* | ABC-type trehalose transporter, substrate-binding lipoprotein | -2.55 |
| *cg0842* |  | Putative DNA helicase | -1.43 |
| *cg0843* |  | Putative helicase | -1.12 |
| *cg0845* |  | DNA/RNA helicase, superfamily II | -1.23 |
| *cg0858* |  | Putative secreted protein | -1.89 |
| *cg0878* | *whcE* | Transcriptional regulator, WhiB-family | -1.03 |
| *cg0881* | *rhlE* | Putative ATP-dependent RNA helicase | -1.22 |
| *cg0898* | *pdxS* | pyridoxal 5'-phosphate (PLP) synthase subunit | -5.01 |
| *cg0899* | *pdxT* | pyridoxal 5'-phosphate (PLP) synthase subunit, glutamine amidotransferase | -5.65 |
| *cg0914* | *ftsE* | Cell division ATP-binding protein FtsE-family | -1.23 |
| *cg0915* | *ftsX* | Putative cell division protein, FtsX-family | -1.33 |
| *cg0921* |  | Siderophore-interacting protein | -1.98 |
| *cg0922* |  | ABC-type putative iron-siderophore transporter, substrate-binding lipoprotein | -1.38 |
| *cg0932* |  | Putative membrane protein | -1.37 |
| *cg0936* | *rpf1* | RPF-protein precursor | -3.13 |
| *cg0948* | *serC* | Phosphoserine transaminase, AT class IV | -1.53 |
| *cg0952* |  | Putative integral membrane protein | -2.05 |
| *cg0953* | *mctC* | secondary acetate/propionate/pyruvate transporter | -1.93 |
| *cg0955* |  | Putative secreted protein | -2.08 |
| *cg0980* |  | Putative secreted protein, related to metalloendopeptidases | -1.78 |
| *cg0988* | *rpsR* | 30S ribosomal protein S18 | -1.21 |
| *cg0989* | *rpsN* | 30S ribosomal protein S14 | -1.19 |
| *cg0990* | *rpmG* | 50S ribosomal protein L33 | -1.25 |
| *cg0991* | *rpmB* | 50S ribosomal protein L28 | -1.24 |
| *cg0994* | *rpmE* | Putative 50S ribosomal protein L31 | -1.6 |
| *cg0995* | *rpmF* | 50S ribosomal protein L32 | -1.33 |
| *cg1002* |  | Conserved putative secreted protein | -1.09 |
| *cg1014* | *pmt* | Protein O-mannosyltransferase | -1.77 |
| *cg1037* | *rpf2* | RPF2 precursor, secreted protein | -1.66 |
| *cg1052* | *cmt3* | Corynomycolyl transferase | -1.21 |
| *cg1072* | *rplY* | Ribosomal protein L25 (general stress protein Ctc) | -1.77 |
| *cg1075* | *prsA* | Ribose-phosphate diphosphokinase | -1.22 |
| *cg1076* | *glmU* | Putative UDP-N-acetylglucosamine diphosphorylase | -1.09 |
| *cg1089* |  | ABC-type putative multidrug transporter, ATPase and permease subunit | -1.02 |
| *cg1090* | *ggtB* | Putative gamma-glutamyltranspeptidase | -2.14 |
| *cg1091* |  | Hypothetical protein | -1.88 |
| *cg1108* |  | Putative secreted protein | -2.7 |
| *cg1109* |  | Hypothetical protein | -1.55 |
| *cg1129* | *aroF* | Putative phospho-2-dehydro-3-deoxyheptonate aldolase | -2.25 |
| *cg1134* | *pab* | Para-aminobenzoate synthase | -1.67 |
| *cg1157* | *fbp* | Fructose-1,6-bisphosphatase | -1.17 |
| *cg1171* |  | Putative GTPase | -1.34 |
| *cg1215* | *nadC* | Putative nicotinate-nucleotide pyrophosphorylase | -1.03 |
| *cg1216* | *nadA* | Quinolinate synthetase, subunit A | -1.49 |
| *cg1241* |  | conserved hypothetical protein - putative pseudogene (C-terminal fragment) | -1.49 |
| *cg1246* |  | Conserved hypothetical protein | -1.01 |
| *cg1247* |  | Putative secreted protein | -1.55 |
| *cg1272* | *cseE* | Anti-sigma factor CseE | -2.93 |
| *cg1298* | *cydC* | ABC-type putative multidrug/protein/lipid transporter, ATPase and permease subunit | -3.29 |
| *cg1299* | *cydD* | ABC-type putative multidrug/protein/lipid transporter, ATPase and permease subunit | -3.48 |
| *cg1300* | *cydB* | Cytochrome d ubiquinol oxidase subunit II | -3.22 |
| *cg1301* | *cydA* | Cytochrome d ubiquinol oxidase subunit I | -2.56 |
| *cg1307* |  | DNA/RNA helicase, superfamily II | -1.27 |
| *cg1313* |  | Putative secreted lipoprotein | -2.37 |
| *cg1314* | *putP* | Putative Na+/proline symporter, solute:sodium symporter (SSS) family | -1.2 |
| *cg1332* |  | Putative secreted hydrolase | -2.33 |
| *cg1340* | *arnR* | transcriptional regulator ArsR-family | -1.81 |
| *cg1341* | *narI* | Respiratory nitrate reductase 2, gamma chain | -1.84 |
| *cg1342* | *narJ* | Respiratory nitrate reductase 2, delta chain | -1.1 |
| *cg1348* |  | Putative membrane protein, containing a CBS domain | -1.03 |
| *cg1354* | *rho* | Transcription termination factor Rho | -1.94 |
| *cg1356* |  | Putative rRNA or tRNA methylase | -1.76 |
| *cg1360* |  | Putative membrane protein | -1.34 |
| *cg1362* | *atpB* | ATP synthase F0, A chain | -1.3 |
| *cg1363* | *atpE* | ATP synthase F0, C chain | -2.19 |
| *cg1364* | *atpF* | ATP synthase F0, B chain | -2.03 |
| *cg1365* | *atpH* | ATP synthase F1, delta subunit | -2.37 |
| *cg1366* | *atpA* | ATP synthase F1, alpha chain | -2.68 |
| *cg1367* | *atpG* | ATP synthase F1, gamma chain | -2.82 |
| *cg1368* | *atpD* | ATP synthase F1, beta chain | -2.49 |
| *cg1373* |  | Putative glyoxalase | -1.74 |
| *cg1394* | *speE2* | Putative spermidine synthase | -1.37 |
| *cg1395* |  | Conserved hypothetical protein | -1.25 |
| *cg1418* |  | ABC-type putative iron-siderophore transporter, substrate-binding lipoprotein | -1.92 |
| *cg1419* |  | Putative secondary Na+/bile acid symporter, bile acid:Na+ symporter (BASS) family | -1.44 |
| *cg1432* | *ilvD* | Dihydroxy-acid dehydratase | -1.77 |
| *cg1435* | *ilvB* | Acetohydroxy acid synthase (AHAS), large subunit | -2.13 |
| *cg1436* | *ilvN* | Acetohydroxy acid synthase (AHAS), small subunit | -2.68 |
| *cg1458* |  | Putative hydrolase, FAA-family | -1.62 |
| *cg1459* |  | Putative SAM-dependent methyltransferase | -1.67 |
| *cg1479* | *glgP1* | Putative glycogen phosphorylase | -3.58 |
| *cg1483* |  | Putative membrane protein | -1.84 |
| *cg1487* | *leuC* | 3-Isopropylmalate dehydratase, large subunit | -1.3 |
| *cg1502* |  | ABC-type putative amino acid transporter, ATPase subunit | -1.05 |
| *cg1513* | *tnp23a(ISCg23a)* | Transposase, putative pseudogene | -1.91 |
| *cg1514* |  | Secreted protein | -2.18 |
| *cg1537* | *ptsG* | Phosphotransferase system (PTS), glucose-specific enzyme IIBCA component | -1.14 |
| *cg1563* | *infC* | Translation initiation factor IF-3 | -1.26 |
| *cg1564* | *rpmI* | 50S ribosomal protein L35 | -1.57 |
| *cg1565* | *rplT* | 50S ribosomal protein L20 | -1.43 |
| *cg1574* | *pheS* | Phenylalanyl-tRNA synthetase, alpha chain | -1.05 |
| *cg1575* | *pheT* | Phenylalanyl-tRNA synthetase, beta chain | -1.27 |
| *cg1585* | *argR* | Transcriptional repressor of arginine biosynthesis, ArgR-family | -1.35 |
| *cg1612* |  | Putative acetyltransferase | -2.46 |
| *cg1614* | *scpB* | Putative segregation and condensation protein B | -1.33 |
| *cg1640* |  | Putative membrane protein, containing a CBS domain | -1.03 |
| *cg1698* | *hisG* | ATP phosphoribosyltransferase | -1.53 |
| *cg1710* | *bacA* | Putative undecaprenol kinase | -1.27 |
| *cg1712* | *lppL* | Putative secreted lipoprotein | -2.25 |
| *cg1713* | *pyrD* | Dihydroorotate oxidase | -2.62 |
| *cg1715* |  | Hypothetical protein | -2.01 |
| *cg1716* | *tnp16b(ISCg16b)* | Transposase | -1.18 |
| *cg1724* |  | Putative protein kinase, ArgK or related GTPase of G3E-family | -1.05 |
| *cg1737* | *acn* | Aconitate hydratase | -1.64 |
| *cg1753* |  | ABC-type transporter, ATPase subunit with duplicated ATPase domain | -2.43 |
| *cg1767* |  | ABC-type putative multidrug transporter, ATPase subunit | -1.36 |
| *cg1769* | *ctaA* | Cytochrome oxidase assembly protein | -1.1 |
| *cg1773* | *ctaB* | Polyprenyltransferase, cytochrome oxidase assembly factor | -1.15 |
| *cg1774* | *tkt* | Transketolase | -1.24 |
| *cg1776* | *tal* | Transaldolase | -1.55 |
| *cg1778* | *zwf* | Glucose-6-phosphate 1-dehydrogenase | -1.31 |
| *cg1779* | *opcA* | Glucose-6-phosphate 1-dehydrogenase subunit | -1.1 |
| *cg1813* | *carB* | Carbamoyl-phosphate synthase, large chain | -1.65 |
| *cg1814* | *carA* | Carbamoyl-phosphate synthase, small chain | -1.89 |
| *cg1824* | *nusB* | Transcription termination factor | -1 |
| *cg1825* | *efp* | Elongation factor P (EF-P) | -1.11 |
| *cg1827* | *aroB* | 3-Dehydroquinate synthase | -1.15 |
| *cg1828* | *aroK* | Shikimate kinase | -1.21 |
| *cg1829* | *aroC* | Chorismate synthase | -1.14 |
| *cg1832* |  | ABC-type putative iron-siderophore transporter, substrate-binding lipoprotein | -1.1 |
| *cg1833* |  | ABC-type putative iron-siderophore transporter, ATPase subunit | -1.78 |
| *cg1834* |  | ABC-type putative iron-siderophore transporter, permease subunit | -2.9 |
| *cg1835* | *aroE3* | Shikimate 5-dehydrogenase | -1.71 |
| *cg1841* | *aspS* | Aspartyl-tRNA synthetase | -1.34 |
| *cg1843* |  | Superfamily II DNA/RNA helicase, SNF2 family | -2.02 |
| *cg1846* |  | Putative transcriptional regulator, TetR-family | -1.72 |
| *cg1848* |  | Putative N5,N10-methylene tetrahydromethanopterin reductase or related flavin-dependent reductase | -1.55 |
| *cg1857* | *ppiB* | Peptidyl-prolyl cis-trans isomerase | -1.29 |
| *cg1859* |  | Putative secreted protein | -1.29 |
| *cg1867* | *secD* | Preprotein translocase, SecD subunit | -1.28 |
| *cg1876* |  | Glycosyltransferase | -1.1 |
| *cg1905* |  | Hypothetical protein | -1.15 |
| *cg1906* |  | Hypothetical protein | -1.24 |
| *cg1907* |  | Putative phosphopantothenoylcysteine synthetase/decarboxylase | -1.38 |
| *cg1908* |  | Hypothetical protein | -1.32 |
| *cg2037* |  | Conserved hypothetical protein | -1.38 |
| *cg2064* |  | DNA topoisomerase | -2.16 |
| *cg2092* | *sigA* | RNA polymerase sigma factor rpoD (Sigma-A) | -1.85 |
| *cg2097* |  | Putative DNA or RNA helicase, superfamily II | -1.2 |
| *cg2103* | *dtxR* | Transcriptional regulator, DtxR-family | -1.15 |
| *cg2104* | *galE* | UDP-glucose 4-epimerase | -1.37 |
| *cg2118* |  | Transcriptional regulator protein, DeoR-family | -1.33 |
| *cg2119* | *fruK* | 1-Phosphofructokinase | -1.71 |
| *cg2120* | *ptsF* | Phosphotransferase system (PTS), fructose-specific enzyme IIABC component | -1.51 |
| *cg2133* |  | Acetyltransferase, GNAT-family | -1.11 |
| *cg2135* | *miaB* | tRNA methylthiotransferase | -1.58 |
| *cg2136* | *gluA* | ABC-type glutamate transporter, ATPase subunit (TC 3.A.1.3.9) | -2.13 |
| *cg2137* | *gluB* | ABC-type glutamate transporter, substrate-binding lipoprotein (TC 3.A.1.3.9) | -2.87 |
| *cg2138* | *gluC* | ABC-type glutamate transporter, permease subunit (TC 3.A.1.3.9) | -3.4 |
| *cg2139* | *gluD* | ABC-type glutamate transporter, permease subunit (TC 3.A.1.3.9) | -3.12 |
| *cg2145* |  | Conserved hypothetical protein | -1.18 |
| *cg2155* |  | Conserved hypothetical protein | -1.02 |
| *cg2167* | *rpsO* | 30S ribosomal protein S15 | -1.57 |
| *cg2170* | *truB* | Pseudouridylate synthase | -1.25 |
| *cg2176* | *infB* | Translation initiation factor 2 (GTPase) | -1.06 |
| *cg2181* |  | ABC-type putative dipeptide/oligopeptide transporter, substrate-binding lipoprotein | -1.56 |
| *cg2183* |  | ABC-type putative dipeptide/oligopeptide transporter, permease subunit | -1.13 |
| *cg2184* |  | ABC-type putative dipeptide/oligopeptide transporter, ATPase subunit | -1.09 |
| *cg2202* |  | ABC-type transporter, permease subunit | -2.79 |
| *cg2204* |  | ABC-type transporter, ATPase and permease subunit | -2.26 |
| *cg2230* | *rnhB* | Ribonuclease HII | -1.89 |
| *cg2240* | *thiF* | Putative Dinucleotide-utilizing enzyme involved in thiamine biosynthesis | -3.11 |
| *cg2241* | *tex* | transcriptional accessory protein Tex | -1.47 |
| *cg2251* | *rimM* | Putative 16S rRNA processing protein, RimM-like | -1.04 |
| *cg2253* | *rpsP* | 30S ribosomal protein S16 | -2.33 |
| *cg2272* | *mutM1* | Formamidopyrimidine-DNA glycosylase | -1.06 |
| *cg2308* |  | Putative secreted protein | -1.24 |
| *cg2320* |  | Putative transcriptional regulator, ArsR-family | -1.56 |
| *cg2336* |  | Putative secreted protein | -1.04 |
| *cg2345* |  | Putative secreted protein | -1.25 |
| *cg2348* |  | Putative secreted protein | -1.16 |
| *cg2402* |  | Secreted protein NLP/P60 family | -3.28 |
| *cg2414* | *cobT* | Nicotinate-nucleotide--dimethylbenzimidazole phosphoribosyltransferase | -1.15 |
| *cg2415* | *cobS* | Adenosylcobinamide-GDP ribazoletransferase | -1.58 |
| *cg2418* | *ilvE* | Branched-chain amino acid aminotransferase, AT class III | -1.19 |
| *cg2425* |  | Putative permease | -1.22 |
| *cg2438* |  | Hypothetical protein | -1.93 |
| *cg2440* |  | Putative sugar/metabolite permease, MFS-type | -1.37 |
| *cg2507* |  | Putative membrane protein | -1.68 |
| *cg2523* | *malQ* | 4-Alpha-glucanotransferase | -1.04 |
| *cg2524* |  | ABC-type putative multidrug transporter, ATPase and membrane subunit | -2.3 |
| *cg2538* |  | Alkanal monooxygenase (FMN-linked) | -1 |
| *cg2556* |  | Putative integral membrane protein | -2.75 |
| *cg2559* | *aceB* | Malate synthase | -2.19 |
| *cg2560* | *aceA* | Isocitrate lyase | -2.47 |
| *cg2565* |  | Hypothetical protein | -1.16 |
| *cg2566* |  | Putative secreted protein | -1.22 |
| *cg2573* | *rpsT* | 30S ribosomal protein S20 | -1.68 |
| *cg2592* |  | Hypothetical protein | -1.43 |
| *cg2594* | *rpmA* | 50S ribosomal protein L27 | -1.54 |
| *cg2595* | *rplU* | 50S ribosomal protein L21 | -1.77 |
| *cg2603* | *ndk* | Nucleoside-diphosphate kinase | -1.31 |
| *cg2610* |  | ABC-type putative dipeptide/oligopeptide transporter, substrate-binding lipoprotein | -3.84 |
| *cg2623* | *pcaI* | Putative 3-oxoadipate CoA-transferase | -1.22 |
| *cg2630* | *pcaG* | Protocatechuate 3,4-dioxygenase, alpha subunit | -1.05 |
| *cg2631* | *pcaH* | Protocatechuate 3,4-dioxygenase, beta subunit | -1.32 |
| *cg2637* | *benA* | Putative benzoate 1,2-dioxygenase, alpha subunit | -3.25 |
| *cg2639* | *benC* | Putative benzoate 1,2-dioxygenase, electron transfer subunit | -1.83 |
| *cg2642* | *benK* | Putative MFS-type benzoate permease | -2.74 |
| *cg2643* | *benE* | Putative secondary benzoate symporter, benzoate:H+ symporter (BenE) family | -3.13 |
| *cg2647* | *tig* | Putative Trigger factor,invoved in cell division | -1.31 |
| *cg2651* |  | Conserved hypothetical protein, putative pseudogen | -1.15 |
| *cg2658* | *rpi* | Ribose-5-phosphate isomerase | -1.06 |
| *cg2701* |  | Putative membrane protein | -1.09 |
| *cg2703* |  | ABC-type putative sugar transporter, permease subunit | -1.37 |
| *cg2704* |  | ABC-type putative sugar transporter, permease subunit | -1.21 |
| *cg2705* | *amyE* | ABC-type putative sugar transporter, substrate-binding lipoprotein | -1.09 |
| *cg2707* |  | Conserved hypothetical protein | -1.12 |
| *cg2750* |  | Conserved putative membrane protein | -1.46 |
| *cg2778* |  | Conserved hypothetical protein | -2.27 |
| *cg2779* | *serB* | Phosphoserine phosphatase | -1.05 |
| *cg2782* | *ftn* | Ferritin-like protein | -1.81 |
| *cg2838* |  | Putative dithiol-disulfide isomerase | -1.33 |
| *cg2840* | *actA* | Coenzyme A hydrolase/transferase | -1.08 |
| *cg2850* |  | Conserved hypothetical protein | -1.23 |
| *cg2851* |  | Branched-chain amino acid aminotransferase, AT class III/4-amino-4-deoxychorismate lyase | -1.38 |
| *cg2857* | *purF* | Amidophosphoribosyltransferase | -2.04 |
| *cg2862* | *purL* | Phosphoribosylformylglycinamidine synthase subunit | -1.24 |
| *cg2874* | *purC* | Phosphoribosylaminoimidazolesuccinocarboxamide synthase | -1.06 |
| *cg2876* | *purB* | Adenylosuccinate lyase | -2.16 |
| *cg2878* | *purD* | Phosphoribosylamine--glycine ligase | -1.36 |
| *cg2906* |  | Conserved hypothetical protein | -1.55 |
| *cg2907* | *otsA* | Trehalose-6-phosphate synthase | -1.21 |
| *cg2925* | *ptsS* | Phosphotransferase system (PTS), sucrose-specific enzyme IIBCA component | -1.78 |
| *cg2931* | *nanA* | N-acetylneuraminate lyase | -1.35 |
| *cg2937* |  | ABC-type putative dipeptide/oligopeptide transporter, substrate-binding lipoprotein | -3.84 |
| *cg2938* |  | ABC-type putative dipeptide/oligopeptide transporter, permease subunit | -3.47 |
| *cg2939* |  | ABC-type putative dipeptide/oligopeptide transporter, ATPase subunit | -4.09 |
| *cg2940* |  | ABC-type putative dipeptide/oligopeptide transporter, ATPase subunit | -1.97 |
| *cg2945* | *ispD* | Putative 2-C-methyl-D-erythritol 4-phosphate cytidylyltransferase | -1.63 |
| *cg2954* | *cynT* | Carbonate dehydratase | -1.2 |
| *cg2966* |  | Putative phenol 2-monooxygenase | -2.66 |
| *cg2974* | *lysS* | Lysine--tRNA ligase | -1.6 |
| *cg3009* | *porH* | Porin, cation-specific | -1.02 |
| *cg3022* |  | Conserved hypothetical protein | -2.34 |
| *cg3028* | *mrpF* | Putative secondary Na+/H+ antiporter, monovalent cation:proton antiporter-3 (CPA3) family | -1.6 |
| *cg3046* | *pknG* | Serine/threonine protein kinase | -1.29 |
| *cg3075* | *cmr* | Putative multidrug efflux permease, MFS-type | -1.16 |
| *cg3124* |  | Conserved hypothetical protein | -2.5 |
| *cg3125* | *tctA* | citrate uptake transporter, membrane subunit | -1.6 |
| *cg3126* | *tctB* | citrate uptake transporter, membrane subunit | -1.73 |
| *cg3133* |  | ABC-type putative cobalt/sugar transporter, ATPase subunit | -1.84 |
| *cg3138* |  | Band 7 domain-containing protein, stomatin/prohibitin homolog | -1.11 |
| *cg3149* | *alaT* | Putative aspartate aminotransferase, AT class I | -2.18 |
| *cg3161* |  | Putative membrane protein | -1.22 |
| *cg3186* | *cmt2* | Trehalose corynomycolyl transferase | -1.23 |
| *cg3194* |  | Putative membrane-associated PA-phosphatase related phosphoesterase | -1.21 |
| *cg3195* |  | Putative flavin-containing monooxygenase | -2.21 |
| *cg3207* | *pheA* | Prephenate dehydratase | -1.29 |
| *cg3213* |  | Putative secreted protein | -2.63 |
| *cg3214* |  | Conserved hypothetical protein | -1.29 |
| *cg3216* | *gntP* | Putative secondary gluconate symporter, gluconate:H+ symporter (GntP) family | -1.8 |
| *cg3225* |  | Putative serine/threonine-specific protein phosphatase | -2.51 |
| *cg3226* |  | Putative MFS-type L-lactate permease | -1.47 |
| *cg3258* | *rluC2* | Pseudouridylate synthase | -1.79 |
| *cg3267* |  | Putative membrane protein, putative pseudogene (C-terminal fragment) | -4.08 |
| *cg3277* |  | Hypothetical protein, containing double-stranded beta-helix domain | -1.08 |
| *cg3301* |  | Putative sugar/metabolite permease, MFS-type | -1.13 |
| *cg3323* | *ino1* | Inositol-3-phosphate synthase | -2.22 |
| *cg3325* |  | Conserved hypothetical protein | -1.12 |
| *cg3334* |  | Putative sugar permease, MFS-type | -1.65 |
| *cg3346* | *leuS* | Leucine--tRNA ligase | -1.07 |
| *cg3356* |  | Putative secondary H+/Na+:glutamate/dicarboxylate symporter, dicarboxylate/amino acid:cation symporter (DAACS) family | -1.25 |
| *cg3359* | *trpE* | Anthranilate synthase subunit I | -2.56 |
| *cg3360* | *trpG* | Anthranilate synthase subunit II | -2.53 |
| *cg3361* | *trpD* | Anthranilate phosphoribosyltransferase | -1.91 |
| *cg3362* | *trpCF* | Phosphoribosylanthranilate isomerase | -1.57 |
| *cg3363* | *trpB* | Tryptophan synthase beta chain | -1.75 |
| *cg3364* | *trpA* | Tryptophan synthase alpha chain | -1.31 |
| *cg3365* | *rmpC* | Phosphotransferase system (PTS), putative ribitol/mannitol-specific enzyme IIC component | -1.99 |
| *cg3366* | *rmpA* | Phosphotransferase system (PTS), putative ribitol/mannitol-specific enzyme IIAB component | -2.29 |
| *cg3380* |  | Putative oxidoreductase protein | -1.25 |
| *cg3386* | *tcbF* | Maleylacetate reductase | -1.38 |
| *cg3389* |  | Predicted oxidoreductase | -1.06 |
| *cg3390* |  | Putative sugar phosphate isomerase/epimerase | -2.16 |
| *cg3404* |  | ABC-type putative iron(III) dicitrate transporter, substrate-binding lipoprotein | -2.34 |
| *cg3431* | *rnpA* | Ribonuclease P | -1.84 |
| *cg3432* | *rpmH* | 50S ribosomal protein L34 | -1.81 |
| *cg4005* |  | Putative secreted protein | -2.09 |
| *cg4016* | *ilvL* | valine-containing leader peptide | -1.86 |
| *cg4017* | *aroR* | tyrosine and phenylalanine-containing leader peptide | -1.42 |
